# 2D polarization imaging as a low-cost fluorescence method to detect α-synuclein aggregation ex vivo in models of Parkinson’s disease

**DOI:** 10.1038/s42003-018-0156-x

**Published:** 2018-10-02

**Authors:** Rafael Camacho, Daniela Täuber, Christian Hansen, Juanzi Shi, Luc Bousset, Ronald Melki, Jia-Yi Li, Ivan G. Scheblykin

**Affiliations:** 1 Chemical Physics and NanoLund, Lund University, P.O. Box 124,, 22100 Lund, Sweden; 2 Department of Chemistry, KU Leuven, Celestijnenlaan 200F, 3001 Leuven, Belgium; 3 Biopolarisation, Leibniz Institute of Photonic Technology, Albert-Einstein-Str. 9, 07745 Jena, Germany; 4 Institute of Solid State Physics, FSU Jena, Helmholtzweg 3, 07743 Jena, Germany; 5 Neural Plasticity and Repair Unit, Wallenberg Neuroscience Center, Department of Experimental Medical Science, Lund University, BMC A10, 22184 Lund, Sweden; 6 Molecular Neurobiology, Department of Experimental Medical Science, BMC B11, 221 84 Lund, Sweden; 7 Institut Fancois Jacob (MIRCen), CEA and Laboratory of Neurodegenerative Diseases, CNRS, 18 Route du Panorama, 92265 Fontenay-Aux-Roses cedex, France; 8 Institute of Health Sciences, China Medical University, 110122 Shenyang, People’s Republic of China

## Abstract

A hallmark of Parkinson’s disease is the formation of large protein-rich aggregates in neurons, where α-synuclein is the most abundant protein. A standard approach to visualize aggregation is to fluorescently label the proteins of interest. Then, highly fluorescent regions are assumed to contain aggregated proteins. However, fluorescence brightness alone cannot discriminate micrometer-sized regions with high expression of non-aggregated proteins from regions where the proteins are aggregated on the molecular scale. Here, we demonstrate that 2-dimensional polarization imaging can discriminate between preformed non-aggregated and aggregated forms of α-synuclein, and detect increased aggregation in brain tissues of transgenic mice. This imaging method assesses homo-FRET between labels by measuring fluorescence polarization in excitation and emission simultaneously, which translates into higher contrast than fluorescence anisotropy imaging. Exploring earlier aggregation states of α-synuclein using such technically simple imaging method could lead to crucial improvements in our understanding of α-synuclein-mediated pathology in Parkinson’s Disease.

## Abstract (web summary)

Camacho et al. show detection of non-aggregated and aggregated α-synuclein in brain tissue from a mouse model of Parkinson’s disease using 2-dimensional polarization imaging. This fluorescence imaging method will allow for early detection of pathogenic α-synuclein aggregates associated with neurodegenerative illnesses.

## Introduction

The aggregation of specific proteins is linked to different neurodegenerative conditions, such as Alzheimer’s and Parkinson’s diseases^1^. Currently, insight into protein aggregation can be gained from in situ investigations using various imaging methods^2–4^. However, to design better treatments, further information on pathological protein aggregation in the brain is needed. For example, in Parkinson’s disease, the nature of the pathogenic α-synuclein (α-syn) assemblies is still debated, e.g., low (oligomeric) or high (fibrillar) molecular weight assemblies^5–9^. Hitherto, no reliable methods exist to distinguish between fibrils, lower molecular weight assemblies, and non-toxic monomeric α-syn. Therefore, to better understand and treat neurodegenerative diseases it is important to map not only the local concentration of the proteins of interest but more importantly their aggregation state.

The standard approach to visualize aggregation is to label the proteins of interest with a fluorescence marker, e.g., via immunohistochemical approaches. This way, the presence of bright areas in the fluorescence image suggests accumulation and, therefore, aggregation of the labeled molecules into large agglomerates^6,7,10–13^. This criterion is, however, ambiguous, because it cannot distinguish regions with only high protein expression where proteins are populated without actual aggregation from authentic aggregates containing densely packed protein molecules. For making this distinction it is necessary to assess the distance between the protein molecules at scales <10 nm, which is far beyond the resolving power of an optical microscope.

Valuable information on the morphology and aggregation dynamics of α-syn has been obtained using super-resolution techniques such as STED^14,15^, dSTORM^16,17^, PALM^18^, and SIM^17^, which are able to reveal structural details with a resolution of some tens of nanometers^19–25^. These methods require special labeling, often expensive equipment and are time-consuming when millimeter-sized areas are to be analyzed for screening. Even though super-resolution methods revolutionized biology by allowing imaging of a whole new set of structures and interactions at sub-diffraction resolution (500–10 nm), these methods are not suited to estimate distances between biological objects lesser than 10 nm, e.g., the distance between two proteins within an aggregate.

It is at this <10 nm distance range that the phenomenon of Förster resonance energy transfer (FRET) comes into play as a well-established nano-ruler between roughly 2 and 10 nm. Therefore, measuring FRET between labeled proteins is an excellent way to assess their aggregation^26–28^. By FRET an excited fluorophore (donor) can transfer its energy to another molecule (acceptor) leading to its fluorescence. The key to using FRET as a nano-ruler is the dependence of the FRET efficiency on the donor-acceptor distance. For example, FRET applied to α-syn in buffer solution and in live cells allowed the assessment of early aggregation states within the amyloid formation pathway^29–32^.

Using different fluorophores as donor and acceptor (hetero-FRET) allows for spectral separation of the acceptor and donor fluorescence. However, the drawback of hetero-FRET methods is their need for two-color labeling, making them technically difficult and sometimes unpractical. On the other hand, FRET between identical fluorophores (homo-FRET) that are differently oriented in space can be detected by correlating the orientation of the transition dipole moments initially excited with those of the finally emitting molecules. This can be achieved by measuring the fluorescence anisotropy (FA) induced by exciting the sample with linearly polarized light^28,33^.

Homo-FRET leads to a decreased steady-state FA value, *r*, in comparison to so-called fundamental anisotropy, *r*_0_ (e.g., *r*_0_ = 0.4 for parallel absorbing and emitting transition dipole moments in a fluorophore), and a time-decay of FA at the sub-nanosecond timescale when using pulsed excitation^28,33–35^. This fluorescence depolarization can also be induced by rotational diffusion of the fluorophores, which can be prevented by using large slowly rotating fluorophores, such as green fluorescent protein (GFP)^28^. For example, homo-FRET has been applied to probe amyloid formation of α-syn labeled with yellow fluorescent protein in solution^36^.

An important and sometimes forgotten consideration when using FA is that its value *r* can only be correctly related to FRET efficiency for isotropically absorbing samples. This is because the direction of the excitation polarization and the two polarizations probed in emission (parallel and perpendicular to the excitation electric field direction, *r* = (*I*_||_ − *I*_⊥_)/(*I*_||_ + 2*I*_⊥_)) are fixed to the laboratory frame. However, structures with anisotropic absorption can possess an arbitrarily oriented alignment axis. Therefore, the fundamental anisotropy (measured in the absence of FRET and molecular rotations) depends not only on the degree of alignment (characterized by linear dichroism), but also on the orientation of the alignment axis relative to the laboratory frame (details in Supplementary Note 1). Furthermore, for such oriented samples, the value of FA in the case of complete energy transfer is not zero^37^.

To avoid anisotropy in absorption, samples are labeled in a way that the label’s orientation has no correlation with the target biological structure (e.g., the membrane plane, etc.). This, of course, limits the type of labels used and eliminates information about the local structural organization of the sample that could otherwise be obtained using polarization sensitive experiments. However, the presence of a linker between target and label hinders the orientational freedom of the label in one or more axes relative to the target. Furthermore, controlled dimerization of fluorescent proteins has shown that chromophores in dimers and oligomers are often not randomly oriented^28^, which has been attributed to interactions between the fluorescent proteins themselves^28,35^. This shows that accomplishing truly isotropic samples is in practice quite difficult.

In this study, we describe the implementation of 2-dimensional polarization imaging (2D POLIM) for assessing protein aggregation via homo-FRET measurements. 2D POLIM was developed for measuring energy transfer at the single-molecule level, and has shown its potential in material sciences^37–39^. 2D POLIM evaluates homo-FRET by a parameter called energy funneling efficiency (*ε*) that ranges from 0 (absence of FRET) to 1 (100% efficient FRET). Here, we demonstrate its ability to map the degree of aggregation of human α-syn fused with GFP (α-syn-GFP) expressed in the brain of transgenic mice. Thanks to the new homo-FRET image contrast *ε*, we could detect differences in protein aggregation in regions that would otherwise be unnoticed if judged only by their fluorescence intensity or FA. Contrary to FA, in 2D POLIM the fluorescence intensity is measured for many combinations of the excitation and detection polarization directions, obtaining more complete information on the fluorescence polarization of the sample. *ε* is essentially independent of the local degree of chromophore alignment, and therefore is applicable to samples with and without anisotropic absorption. The 2D POLIM methodology does not differ considerably from conventional low-cost wide-field fluorescence microscopy, not requiring pulsed laser sources, time-resolved detection and/or sample scanning.

## Results

### Interpretation of the energy funneling parameter

**Fig. 1:**
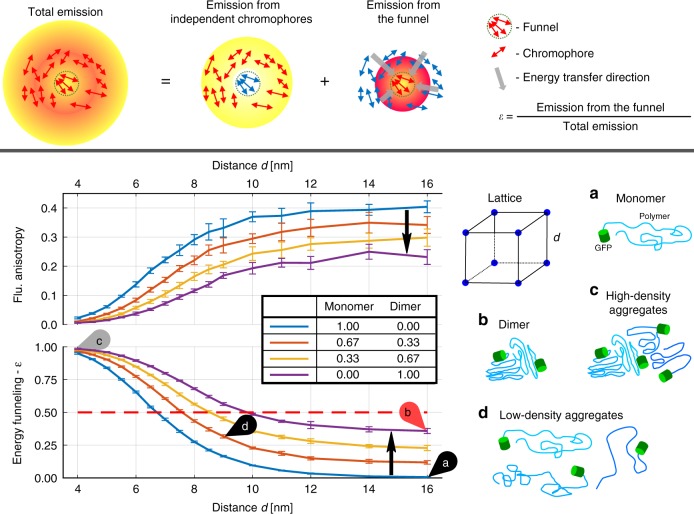
Definition of energy funneling efficiency *ε* (top panel) and calculations of the fluorescence anisotropy r and *ε* as functions of the distance between GFP molecules in a cubic lattice (bottom panel). The homo-FRET Förster radius for GFP is 4.7 nm according to its spectral properties. On the right column pictograms of: **a** monomer, **b** dimer, **c** densely, and **d** loosely packed aggregates of α-syn-GFP. The monomer/dimer ratio indicates how many sites of the lattice are occupied by a monomer/dimer. Black arrows show the transition from the pure monomer to the pure dimer case. Error bars represent the standard deviation of the simulations when repeated 10 times

Throughout this work we implement a new FRET efficiency parameter called energy funneling efficiency—*ε*. Due to the novelty of our approach, we refer readers interested in the exact definition of ε to the Method’s section, Supplementary Note 1 and previously published papers^40,41^. The main idea behind *ε* is illustrated by a scheme in Fig. 1 (top). Fluorescence of any multi-chromophoric system can be split into two components: the light coming from chromophores initially excited and the light coming from chromophores indirectly excited via energy transfer from the light absorbing chromophores. It was proposed to approximate this general situation via the so-called single funnel approximation assuming FRET to only one common set of chromophores called energy funnel or common acceptor. The emission contribution of the funnel (acceptor in FRET terms) to the total emission of the system is called the energy funnelling parameter *ε*.

In order to use the energy funneling parameter, *ε* (FRET efficiency parameter), as a ruler at the nanoscale we must know the dependence between *ε* and the distance between fluorescent labels. Further, *ε* should be directly compared to fluorescence anisotropy, which is a more common parameter for assessing homo-FRET. For this purpose, we made a series of simulations where we calculated the FA and *ε* parameter for GFP molecules at different inter-chromophoric distances, where excitation energy transfer among chromophores was modeled via classical Förster theory (details presented in the Methods section). Briefly, we placed randomly oriented GFP molecules in a cubic lattice and allowed for either radiative decay or FRET to another molecule.

As one can see from Fig. 1, FA presents a well-known decrease with decreasing inter-chromophoric distance: (i) *r* = 0.4 when the distance *d* between GFP molecules is large enough that no FRET occurs among them (*d* > 12 nm, and thus considerably larger than the Förster radius *R*_0_ = 4.7 nm), (ii) *r* has an approximately linear dependence, monotonically decreasing with distance for 9 nm > *d* > 5 nm, (iii) *r* = 0 when *d* < 4 nm. The decrease in FA is a consequence of energy transfer among differently oriented chromophores, which decreases the emission polarization of the ensemble in comparison to the initially photoselected state generated by the linearly polarized excitation light.

In contrast, the energy funneling parameter presents an increase with decreasing distance *d*: (i) *ε* ≈ 1 for *d* < 4 nm, (ii) *ε* has an approximately linear dependence with distance for 5 nm < *d* < 9 nm, and (iii) *ε* = 0 for *d* > 12 nm. Despite the simplicity of this model, it gives us a good idea about the length scale in which FA and *ε* are sensitive to FRET.

Due to the complexity of the problem, we can only provide a numerical solution to the homo-FRET process in a lattice of finite size. Therefore, it is expected that the simulation yields slightly different results when repeated. This is the origin of the error bars presented in Fig. 1. Interestingly, we observe that *ε* values are considerably more reproducible/robust (smaller error bars) than FA. One reason for this is that accomplishing fully isotropic dipole systems numerically is difficult without using a number of dipoles that would render computational times prohibitively long. Therefore, the amplitude of the FA error bars partly reflects the sensitivity of the FA to orientation artifacts. We explore this point in more details in Supplementary Note 1 and Supplementary Figure 2, and we will come back to it from an experimental point of view at the end of our discussion and in Supplementary Discussion 3.

Let us expand the discussion of these results within the context of α-syn aggregation. First, we need to understand how large the GFP molecule and α-syn protein are. For α-syn (140 amino acids) this is a challenging issue because the size of the protein coil depends on environmental conditions. For example, fully collapsed α-syn has a radius of gyration of about 4 nm, while an extended conformation of the protein can be easily several tens of nanometers long (53 nm for fully extended α-syn)^42^. In the case of GFP, its barrel, which contains the actual chromophore in its center, has a diameter of about 3 nm with a length of about 4 nm^26,28,43^.

Considering these characteristic sizes, densely packed α-syn molecules provide conditions for GFP molecules to be separated on average by distances *d* < 7 nm leading to *ε* ≥ 0.5. For example, if we assume that fully collapsed α-syn chains are aggregated, then the characteristic distance between GFPs would be about 8 nm (two times the radius of gyration). Of course, particular conformations can allow even smaller distances and, therefore, a dense aggregate of α-syn should have *ε* > 0.5.

On the other hand, for FRET to occur only two molecules are required. However, such protein dimer is not what researchers usually refer to when dealing with protein aggregation. The question thus is how large we expect the *ε* signature to become for α-syn dimers when they are still far away from each other. To answer this question, we replaced a fraction of the monomers in the previously discussed lattice model with dimers of randomly oriented GFP molecules. This calculation shows (Fig. 1) that the presence of dimers indeed leads to some value of *ε* which is smaller than 0.35 when the dimers are far away from each other. For distances between the dimers in the range of 4–10 nm, the behavior of *ε* remains similar to that of the monomers only situation, particularly while the monomer-dimer ratio remains below 0.5. Therefore, we can conclude that *ε* > 0.5 means that the α-syn protein is densely aggregated and the size of the aggregates exceeds two protein molecules.

Moreover, the FA of GFP dimers is often larger than that expected if the chromophores in the dimers were randomly oriented^28^. It has been suggested that this preferential collinearity between chromophores in GFP dimers is due to interactions between the fluorescent proteins themselves^28,35^. In terms of our simulations, this would mean that the energy funneling increase (FA decrease) due to the presence of dimers would be smaller than that presented in Fig. 1b. Therefore, we can consider the results presented in Fig. 1 as an upper limit for the influence of dimers and small oligomers to *ε* values (see Supplementary Discussion 5 for details).

Note that the simulations discussed here are valid for any other non-rotating fluorescence label, not only GFP. For any other dye, if the homo-FRET Förster radius is similar to that of GFP, then the results are numerically the same, while changes in Förster radius would only shrink or stretch the distance axis. On the other hand, rotational diffusion of the labels would increase the epsilon values for no-FRET conditions (*d* ≫ Förster radius) to a level that depends on the ratio between the fluorescence lifetime and the rotational correlation time. Such effect is similar to that observed for the presence of dimers (Fig. 1b).

### Monomers and fibrils of α-synuclein in cells

To determine experimentally if the parameter *ε* is able to discern between the monomeric and aggregated forms of α-syn, we performed 2D POLIM experiments on neuronal MN9D cells, which had been incubated with either monomeric or preformed fibrillar forms of α-syn (transmission electron images available in Supplementary Figure 16)^44,45^. α-syn was labeled with ATTO-550 (homo-FRET Förster radius: *R*_0_ = 5.6 nm), where, on average, each α-syn contained more than one dye. When many α-syn molecules form a dense aggregate (e.g., beta-sheet structures) the distance *d* between fluorescent labels becomes smaller than 8 nm allowing for homo-FRET between the dye labels (Fig. 1). The larger the density of proteins in the aggregates, the larger the efficiency of the FRET processes. Therefore, *ε* is expected to be larger in α-syn fibrils than in the monomeric form of the protein, as demonstrated by the aforementioned simulations.

**Fig. 2:**
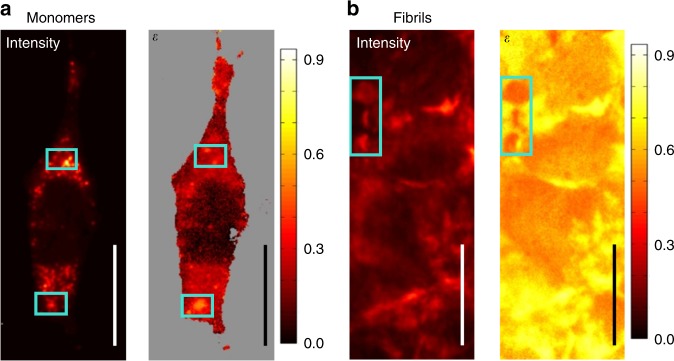
Fluorescence intensity and energy funneling efficiency for preformed α-syn assemblies. **a** monomers and **b** fibrils of α-syn labeled with ATTO-550 and incubated in MN9D cells. Boxed regions highlight differences between intensity and *ε* contrasts. Scale bars are 26 μm long

α-syn monomers and fibrils successfully entered the cells and settled into their cytoplasm (Fig. 2)^45^. In both cases, the distribution of α-syn in the cell’s cytoplasm was uneven. While monomeric α-syn molecules formed bright fluorescent clusters smaller than the diffraction limit, fibrils accumulated in structures larger than several micrometers. The fluorescence intensity showed that, as expected, the amount of α-syn in the fibrillar structures was considerably larger than that in the clusters of monomers.

Most importantly, the images in energy transfer contrast show that *ε* in the fibrillar structures was significantly larger (*ε* = 0.65 ± 0.08) than in the monomer’s case (*ε* = 0.2 ± 0.1, see Supplementary Table 1 and Supplementary Discussion 2), which is consistent with our simulations (Fig. 1). This demonstrates experimentally that *ε* is a suitable parameter to detect α-syn aggregation, allowing us to distinguish between preformed densely packed fibrils and loose monomer clusters formed in the cells.

In simple fluorescence assays, aggregates of labeled proteins are identified by their bright fluorescence due to the high density of the label molecules. However, this simple optical method cannot tell the difference between areas of low and high density at the nm level. In both cases, many proteins can be located within a small volume (hundreds of nm) leading to a bright spot in the fluorescence image. The boxed regions in Fig. 2 illustrate this problem and detailed correlation plots between fluorescence intensity and *ε* can be found in Supplementary Figures 5–6. In Fig. 2a, one can see bright spots in the fluorescence intensity image, which do not show any elevated *ε*, indicating that these are not densely packed aggregates of α-syn monomers. Similarly, Fig. 2b shows that some highly luminescent fibrillar structures had in fact lower FRET signature, *ε* ≈ 0.4, than average, *ε* ≈ 0.7, which suggests that the density of the fibrils is not constant. In other words, some fibrils, despite their elevated fluorescence intensity, were not as densely packed as others, and as consequence the distance between α-syn was large enough to hinder the FRET process. These are perfect examples of how 2D POLIM gives structural information, which is not possible to obtain via conventional fluorescence imaging.

### α-synuclein aggregation in the mouse brain

The α-syn-GFP transgenic mouse model studied in our current report has been previously investigated via two-photon fluorescence microscopy. These studies revealed large expression of α-syn-GFP (high fluorescence intensity of GFP) in several brain regions, and demonstrated increased aggregation with increasing mouse age^12,13,46^. While the GFP tag is larger than α-syn and therefore may alter the rate of α-syn aggregation, it does not prevent aggregation as shown in many studies^10–12^. Further, studies using yellow fluorescent protein (YFP) fused to α-syn showed that amyloids formed by α-syn-YFP are essentially identical to the wild-type amyloids formed by α-syn itself^36^. Here we applied 2D POLIM to the olfactory bulb (OB) of α-syn-GFP mice, comparing brain tissue from old (2 years) and young (3 months) mice in order to determine whether 2D POLIM is able to detect α-syn aggregation.

**Fig. 3:**
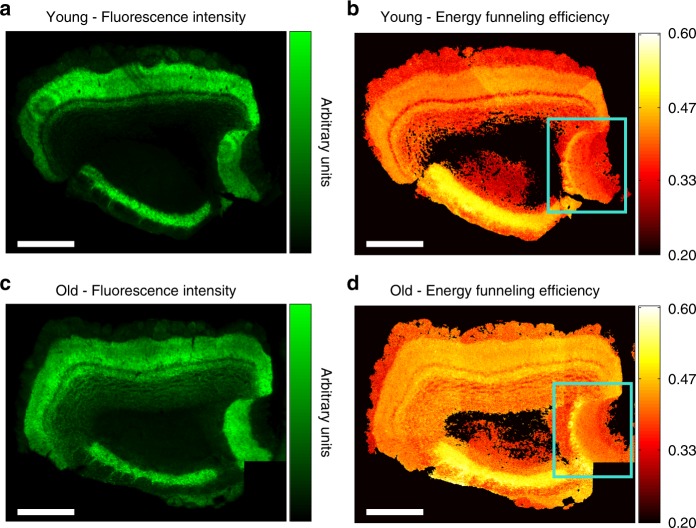
Fluorescence and energy funneling efficiency (*ε*) overview images of the olfactory bulb. OB sections from a young (**a**, **b**) and old (**c**, **d**) mouse for comparison. The signal is generated by fluorescence of α-syn-GFP. On average, a significantly higher energy funneling efficiency is evident in the older mouse (Pearson’s test of independence, *p* < 0.001). Boxed regions highlight the accessory olfactory bulb where important changes occur in the aggregation patterns of young vs. old mice. Scale bars are 0.5 mm long

Overviews of the OB of a representative old and young mouse are presented in Fig. 3. As expected due to their age, the general level of expression of α-syn-GFP was larger in the older mice as we observed from fluorescence intensity images. Further insights are obtained by comparing the *ε* images. A higher average FRET efficiency was observed for the older mice. In particular, the old mice presented a mean *ε* value of 0.43 ± 0.05, while in the young mice the mean *ε* values was 0.33 ± 0.06, pointing to a higher packing density of α-syn-GFP in the older mice (*ε* distributions and Pearson’s test of independence, *p* < 0.001, can be found in Supplementary Tables 2-3 and Supplementary Discussion 3). Moreover, a clearly different aggregation pattern is seen, when comparing the accessory olfactory bulb (AOB) of young and old mice (boxed regions in Fig. 3) as will be discussed below.

**Fig. 4:**
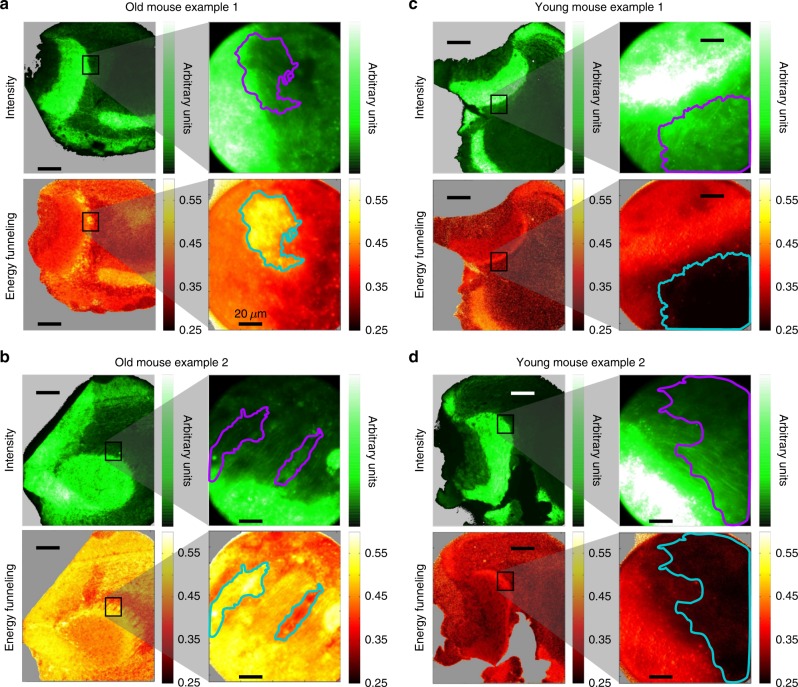
Accessory olfactory bulbs images at low and high magnification. The figure presents two old (**a**, **b**) and two young (**c**, **d**) mice showing high expression of α-synuclein-GFP. Higher magnification images are presented on the right column of each panel. In some areas of the old mice anti-correlation between fluorescence intensity and *ε* is clearly visible while this is absent in the same areas of the young mice AOB. Contours lines are added in the higher magnification images to guide the eye so the correlation between fluorescence intensity and energy funneling efficiency is clearer. Contour lines are calculated using the *ε* image, where all values inside the contour line are larger (or smaller) than an arbitrarily chosen threshold. Scale bars are 200 and 20 μm long for the low and high magnification images, respectively

Higher magnification images of the AOB were obtained (Fig. 4) to investigate the differences between the aggregation pattern of young and old mice in more detail. They reveal local regions of dense α-syn aggregation (*ε* > 0.5) in the AOB of the old mice that were absent in the AOB of the young mice (Fig. 4c, d, and Supplementary Table 4). Further, while in the young mice large FRET efficiency strongly correlated with large fluorescence intensity (Supplementary Figures 7, 12, and 13), an anti-correlation between fluorescence intensity and FRET efficiency was often observed in the old mice (Fig. 4a, b, and Supplementary Figures 8, 10, 11).

As we have mentioned before, high fluorescence intensity is not necessarily a sign of protein aggregation. Moreover, when chromophores are densely packed so-called concentration-dependent fluorescence quenching^47^ can occur making dense aggregates actually weakly emissive, which again renders the intensity criteria unreliable. However, the *ε* contrast allows detecting such regions of very densely packed proteins. While a complete picture is still elusive, we suggest that the regions of low fluorescence intensity and high *ε* are those where α-syn is aggregated most densely.

### Energy funneling parameter vs fluorescence anisotropy

**Fig. 5:**
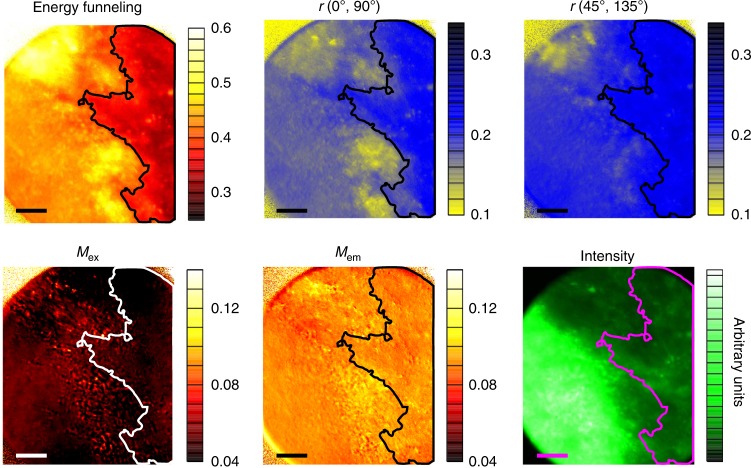
Comparison of different imaging contrasts for an AOB section. The image presents the energy funneling efficiency *ε*, fluorescence anisotropy *r*, modulation depths in excitation and emission (*M*_ex_ and *M*_em_, respectively) and fluorescence intensity of the AOB from an old mouse. Fluorescence anisotropy was calculated for two different orientations of the excitation light polarization (0° and 45°), the detection analyzer then was set to (0°, 90°) and to (45°, 135°), respectively. Note that these two images in the *r*-contrast differ substantially from each other and from the *ε*-image. Contour lines are added as eye guides so differences between imaging contrast are clearer. Contour lines are calculated using the *ε* image, where all values inside the contour line are smaller than 0.35. Scale bars are 20 μm long

As mentioned earlier, traditional FA measurements are not directly applicable for assessing FRET in samples with macroscopic alignment of dipole moments (i.e., presenting linear dichroism), because in that case the FA value depends on the chosen orientation of the excitation light polarization^37^. This is illustrated in Fig. 5 by showing images of an AOB region in different imaging contrasts.

**Fig. 6:**
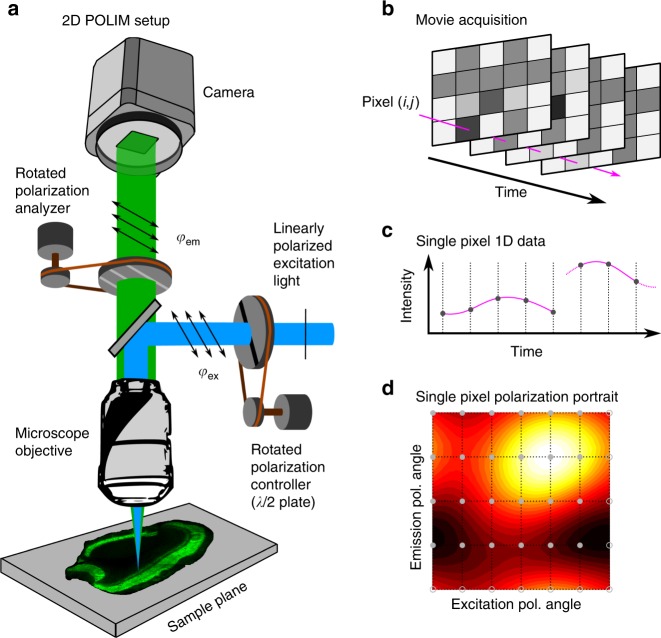
Description of the 2D POLIM methodology. **a** Schematics of the experimental setup. A series of fluorescence images (a movie, **b**) is taken at different combinations of the polarization angles (*φ*_ex_, *φ*_em_). We use 24 images with angles given by the pair permutation of the sets: *φ*_ex_: {0, 30, 60, 90, 120, 150} and φ_em_: {0, 45, 90, 135}. The fluorescence intensity of each pixel (*i*, *j*) as a function of time (**c**) is converted to a 2D plot *I*_*i*,*j*_(*φ*_ex_, *φ*_em_) called polarization portrait (**d**), where *φ*_ex_ and *φ*_em_ are coordinate axes and fluorescence intensity is given by the color code. The analysis of the 2D surface yields several parameters which can be used as imaging contrasts

To image the degree of dipole alignment we use the modulation depth imaging contrasts. The term modulation comes from the fluorescence intensity having a cosine-like dependence on the orientation of the excitation polarization laser and on the orientation of the polarization analyzer installed in front of the detector (Fig. 6, Supplementary Note 1). Modulation depth, *M*, shows the degree of alignment, while the modulation phase, *θ*, shows the main orientation axis for the transition dipole moments responsible for fluorescence excitation (*M*_ex_ and *θ*_ex_,) and fluorescence emission (*M*_em_ and *θ*_em_). Modulation depths are dimensionless quantities with values between 0 (isotropically distributed dipoles) and 1 (uniaxially aligned dipoles). Modulation depths and phases have been widely used in single-molecule/single-particle spectroscopy for assessing structural organization and FRET in individual conjugated polymers chains, molecular aggregates, etc ^37,39–41^.

Although the degree of dipole alignment for the GFP labels in the example was quite low (*M*_ex_, *M*_em_ < 0.12), it was still visible as a fine structure in the *M*_ex_ and *M*_em_ images, and large enough to negatively affect the classical FA measurements. FA images calculated for two different orientations of the excitation light polarizations (vertical and rotated by 45°) differ considerably from each other. Note that this is equivalent to simply rotating the sample on the sample plane, and therefore ideally the images should not differ from one another. Further, the two FA images also differ from the image in energy funneling efficiency (*ε*) contrast. Although FA images show somewhat similar structures as the *ε* image, the latter does not possess the fine structure artifact that correlates with the structures visible in *M*_ex_ and *M*_em_ images.

The effect of macroscopic label orientation on FA is further illustrated in the Supplementary Discussion 4 and Supplementary Figure 14 where 2D POLIM images in all discussed contrasts are shown for a pi-conjugated polymer film. The film, as all films of conjugated polymers, possesses an excellent homo-FRET (large *ε* everywhere) but various degrees of local chain alignment which makes FA images to falsely show regions with poor FRET (large FA). This nicely shows that *ε* is virtually insensitive to the degree of local alignment and gives images of energy transfer efficiency clear from orientation artifacts. Therefore, even for biological samples possessing such small values of linear dichroism, local homo-FRET efficiency can be better estimated by using the energy funneling contrast *ε*.

## Conclusions

We have shown that 2D polarization imaging achieved by a simple modification of a wide-field fluorescence microscope makes it possible to visualize aggregated states of exogenous α-synuclein assemblies labeled with extrinsic fluorophores and GFP-labeled α-synuclein protein in mouse brain tissue with a great certainty. To visualize protein aggregation we employed a special energy funneling parameter that was previously developed for quantitative monitoring of energy funneling in individual chains of conjugated polymers. We showed that strongly fluorescent regions with high expression levels of proteins that are not aggregated can be confidently discriminated from regions where authentic aggregation of the same proteins occurs. Our method is neither sensitive to fluorescence intensity nor to local optical anisotropy, which makes it superior to commonly used techniques relying on local changes of fluorescence intensity or fluorescence anisotropy to detect aggregated proteins. We think that the low cost of the technique, high selectivity, and straightforward data interpretation make it potentially interesting for a wide range of biological research.

## Methods

### Fluorescence imaging—2D polarization imaging method

The 2D POLIM setup is essentially a wide-field fluorescence microscope where fluorescence images are taken for many different orientations of the linearly polarized excitation light (*φ*_ex_) and several different orientations of a polarization analyzer (*φ*_em_) installed in front of the camera (Fig. 6). In this way, the fluorescence intensity *I* of each pixel in the image is recorded as a function of both, *φ*_ex_ and *φ*_em_. From this data we obtain a two-dimensional function, *I*(*φ*_ex_, *φ*_em_) called polarization portrait (Fig. 6d) for each pixel of the image.

The polarization portrait is then used to calculate the 2D POLIM contrasts of each pixel (Supplementary Note 1), such as FA (*r*, see Supplementary Eq. 7), modulation depths in excitation and emission, *M*_**ex**_ and *M*_**em**_ (Supplementary Eqs. 1 and 2), and the energy funneling efficiency, *ε* (FRET efficiency parameter, Supplementary Eqs. 8–15).

Modulations depths depend on the organization of the dipole moments responsible for fluorescence absorption and emission, such as the chromophores of fluorescence labeling assays. Modulation depths are, therefore, closely related to the fluorescence detected linear dichroism (*M*_ex_) and degree of polarization under natural illumination (*M*_em_).

In order to quantify the FRET efficiency of the sample, we analyze the full polarization portrait via the so-called single funnel approximation^40,41^. In this approximation, the polarization portrait is fitted by a model comprised of the linear combination of two components with coefficients (1 − *ε*) and *ε*. The first component of the model assumes the absence of energy transfer and depends solely on the organization of the dipoles responsible for light absorption. On the other hand, the second component assumes the presence of energy transfer towards an effective emitter (subset of emitting states) with fixed polarization properties, such transfer is sometimes referred to as energy funneling (Supplementary Figure 3). The polarization of this emitter can have any character from purely dipolar to completely isotropic. The parameter *ε* ranges from 0, if there is no transfer, to 1, if complete FRET occurs towards the effective emitter. Such definition of the FRET-metric is straightforward and has a clear interpretation at the molecular level. Moreover, the value of *ε* by its definition is not dependent on the degree of dipole alignment making it possible to analyze FRET in samples of varying local linear dichroism.

For additional information about the 2D polarization imaging method please refer to Supplementary Figures 1–4, Supplementary Note 1, and Supplementary Discussion 1–4. Additional supporting information for this study is presented in Supplementary Figures 5–16 and Supplementary Tables 1–4.

### Fluorescence imaging—microscopy setup

2D POLIM experiments were performed on a home-built wide-field fluorescence microscope based on the commercial Olympus IX71 inverted microscope. Samples were excited using an Ar-ion laser. The fibrils and monomers of α-syn labeled with ATTO-550 were excited using 514 nm, while samples containing α-syn-GFP were excited at 488 nm.

The laser light was passed through a suitable clean-up filter before reaching the sample plane. The excitation polarization controller, which consisted of an *λ*/2 achromatic plate (Thorlabs) mounted on a motorized rotation mount (Standa), was used to change the orientation of the linearly polarized light (*φ*_ex_) on the sample plane. The dichroic mirror used to reflect the laser beam was birefringent. Thus a Berek compensator (New Focus) was placed after the excitation controller to preserve the linear polarization of the excitation light at the sample plane. To obtain a smooth sample illumination and eliminate reflections introduced by the optics at the sample plane a spatial filter was used after the polarization controller.

Two objective lenses were used to image the sample: a low magnification ×4 (Olympus, Plan N, NA = 0.1) and a high magnification ×40 (Olympus, LUCPlanFLN, NA = 0.6) giving a pixel size of the EMCCD camera of 2.6 and 0.26 µm when projected to the sample plane, respectively. To avoid depolarization effects in emission, the output port of the microscope was modified to use a mirror instead of a prism to reflect the fluorescence beam towards the detector. The collected fluorescence was passed through an emission analyzer, which controls the emission polarization orientation (*φ*_em_). The emission analyzer consisted of a wire-grid linear polarizer (Edmund optics) mounted in a motorized rotating mount (Standa). Suitable filters were used to further block the laser light scattered by the sample and selectively transmit the fluorescence of the sample. In the case of α-syn labeled with ATTO-550, a 630/69 bandpass filter with transmission in the range 590–670 nm was used. In the case of α-syn labeled with GFP, a longpass filter with edge at 505 nm was used. Finally, the fluorescence emission was imaged on an EMCCD camera (Princeton Instruments, PhotonMax). The motors and the camera were controlled using LabVIEW.

Calibration of polarization sensitive microscopes is not an easy task of paramount importance^48^. The methods we use in our setup are summarized in ref. ^40^ We developed several performance tests routinely done every day before each series of measurements. The first test checks for polarization artifacts both in excitation and emission light paths. It uses a special sample called artificial molecule (test sample that produces fully polarized response, i.e., dipolar, absorption and dipolar emission). The second test measures transmission artifacts (we use an unpolarized test sample, generally a solution of a fluorescent dye).

### Cell culture

MN9D cells were grown in DMEM (Sigma-Aldrich) plus 10% FBS (Sigma-Aldrich) and penicillin/streptomycin (P/S).

### Fluorescent labeling of α-synuclein monomers and fibrils

Monomeric α-synuclein assemblies in buffer A (20 mM Tris, pH 7.5, 150 mM NaCl) was buffer exchanged using NAP10 desalting columns (GE Healthcare) to phosphate-buffered saline (PBS) buffer. We performed α-synuclein labeling with Atto-550 NHS ester fluorophore following the manufacturer’s instructions (Atto-Tec Gmbh) using a protein:label molar ratio of 1:2^44^. The labeling reactions were arrested by the addition of 10 mM Tris pH 7.5. Unreacted fluorophore was removed from monomeric α-synuclein preparations using NAP10 desalting columns.

α-synuclein fibrils were generated as previously described^7^. In short, soluble wild-type α-syn was incubated in buffer A at 37 °C under continuous shaking (Eppendorf Thermomixer, 600 r.p.m). Fibril formation was continuously monitored using a fluorescence spectrometer (Cary Eclipse, Varian Inc., Palo Alto, CA, USA) under stirring (100 r.p.m., magnetic stir bar). in the presence of Thioflavin T (15 mM). The excitation wavelength was set to 440 nm and emissions wavelengths set to 440 and 480 nm, while each time point measurement consisted of an averaging time of 1 s.

For fibrillar α-synuclein labeling, the fibrils were centrifuged twice at 15,000 × *g* for 10 min, resuspended twice in PBS and labeled as described above. The unreacted fluorophore was removed by a final cycle of two centrifugations at 15,000 × *g* for 10 min and resuspension of the pelleted fibrils in PBS^44^. Two Atto molecules on average were incorporated per α-synuclein molecule whether in monomeric or fibrillar form as assessed by MALDI-TOF mass spectrometry analysis.

### Brain sectioning of transgenic mouse model for expression of human α-syn-GFP

BAC-α-synuclein-GFP-transgenic C57BL/6 mice expressing human wild-type α-synuclein fused to GFP were used for this study^12,46^. Brain tissue from homozygous female mice was collected at 24 or 3 months of age. The animals were perfused using 0.9% NaCl followed by 4% PFA and brains were left over night in 4% PFA. Subsequently, the brains were washed with PBS before changing the medium to 20% sucrose for a minimum of one day. Finally, the brains could be sectioned into 30 µm free-floating coronal sections using a microtome. The sections were mounted onto pre-coated glass slides, covered by coverslips and sealed with PVA-DABCO (50%). All work involving animals was approved by the Ethical Committees for use of laboratory animals at Lund University (Jordbruksverket), Sweden.

### Simulations of homo-FRET for randomly oriented GFP molecules/dimers in bulk

Our simulations are a numerical solution to the complex problem of estimating the fluorescence intensity arising from a large ensemble of GFP molecules when excited by polarized light and considering the presence of homo-FRET. The simulation pipeline can be resumed into: (i) generation of a dipole model, (ii) generation of the polarization portrait, and (iii) calculation of the 2D POLIM output from the polarization portrait. The dipole model sets the number, position and orientation of the dipoles. This information together with the spectral properties of the dye is then used to calculate the FRET rate between all dipoles in the system, and then allows us to numerically simulate the process of energy transfer in the multi-chromophoric ensemble.

A dipole model consists of a central dipole surrounded by a large number of buffer dipoles in a cubic lattice, where all dipoles are randomly oriented. The central dipole is the dipole of interest for which the excitation/emission properties will be calculated. The buffer dipoles can be seen as a bath affecting the response of the central dipole depending on their position, orientation and FRET coupling.

This means that for each simulated dipole model the polarization portrait obtained has fully polarized excitation (single absorbing dipole, i.e., central dipole) and an emission polarization that depends on the interaction between the central dipole and its buffer. If the buffer dipoles are far away (tens of nm) then they do not affect the central dipole and emission will also be fully polarized. On the other hand, if the buffer dipoles are very close (<4 nm), then the energy absorbed by the central dipole will be transferred and completely redistributed into the bath, making the emission isotropic. Therefore, to simulate the response coming from a large ensemble of randomly oriented dipoles (e.g., GFP in solution) many (hundreds-thousands) dipole model iterations have to be done and summed together.

To consider the effects of dimers in the polarization properties the program randomly takes a fraction of the sites in the dipole model’s cubic lattice and exchanges the monomers sitting there by model dimers. The model dimer consists of two dipoles with the following properties: (i) the two dipoles are separated by a fixed distance (3.5 nm); (ii) the center of gravity of the model dimer is set to the original cubic lattice position; (iii) the relative orientation of the dipoles inside the dimer is random; (iv) the position of the dipoles relative to their center of mass is also random.

To calculate the excitation energy transfer rate between all dipoles we follow the classical FRET equations^27^. Then by comparing the homo-FRET rate to the fluorescence emission rate, we calculate the probabilities of emission and of transfer to all buffer dipoles. Using this information, we can numerically estimate via an iterative approach the emission of the central dipole after the homo-FRET process has taken place.

### Code availability

The computer code used to generate the data presented in Fig. 1 is publicly available and can be found in https://github.com/CamachoDejay/FRET-calculations. The same website contains detailed explanations of all functions and logic.

## Electronic supplementary material

Supplementary Information

## Data Availability

The datasets generated during and/or analyzed during the current study are available from the corresponding author on reasonable request.
