# Supplementary material for: 2D polarization imaging as a low-cost fluorescence method to detect α-synuclein aggregation ex vivo in models of Parkinson’s disease
Source: Commun Biol. 2018 Oct 2;1:157. doi: 10.1038/s42003-018-0156-x (PMC6168587; doi:10.1038/s42003-018-0156-x)
Supplement: Supplementary file 1 — Supplementary Information [file 42003_2018_156_MOESM1_ESM.pdf]

Supplementary information: 2D polarization imaging as a low-cost fluorescence method to detect α-synuclein aggregation *ex vivo* in models of Parkinson's disease

Rafael Camacho[1,2], Daniela Täuber[1,3,4], Christian Hansen[5,6], Juanzi Shi[1], Luc Bousset[7], Ronald Melki[7], Jia-Yi Li[5,8], Ivan G. Scheblykin[1*a]

[1] Chemical Physics and NanoLund, Lund University, P.O. Box 124, 22100 Lund, Sweden

[2] Department of Chemistry, KU Leuven, Celestijnenlaan 200F, 3001 Leuven, Belgium

[3] Biopolarisation, Leibniz Institute of Photonic Technology, Albert-Einstein-Str. 9, 07745 Jena, Germany

[4] Institute of Solid State Physics, FSU Jena, Helmholtzweg 3, 07743 Jena, Germany

[5] Neural Plasticity and Repair Unit, Wallenberg Neuroscience Center, Department of Experimental Medical Science, Lund University, BMC A10, 22184 Lund, Sweden

[6] Molecular Neurobiology, Department of Experimental Medical Science, BMC B11, 221 84, Lund, Sweden

[7] Institut Fancois Jacob (MIRCen), CEA and Laboratory of Neurodegenerative Diseases, CNRS, 18 Route du Panorama, 92265 Fontenay-Aux-Roses cedex, France

[8] Institute of Health Sciences, China Medical University, 110122 Shenyang, People's Republic of China

Email: *ivan.scheblykin@chemphys.lu.se*

Content:

Supplementary Figures	2
Supplementary Tables	13
Supplementary Notes	15
Supplementary Discussion	19
Correlation between fluorescence intensity and energy funneling	19
Comparison between preformed fibrils and monomers of α-syn	20
Changes in epsilon (aggregation) with mouse age	20
Fluorescence anisotropy vs energy funneling, experimental comparison	20
Extra comments about homo-FRET simulations	21
Supplementary Methods	21
Supplementary References	21

Supplementary Figures

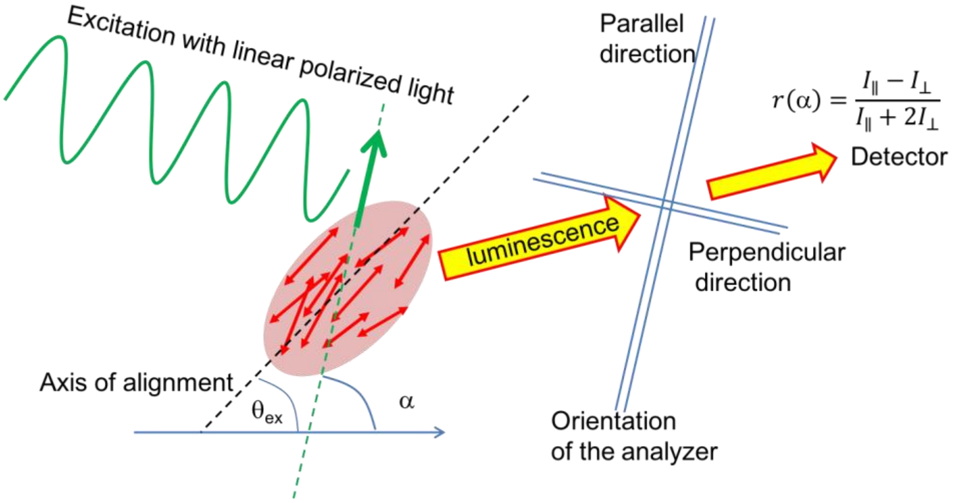

Supplementary Figure 1| Illustration of the fluorescence anisotropy measurements for a system with preferential orientation of dipoles. The orientation of the excitation light (α) determines the orientation of the analyzer.

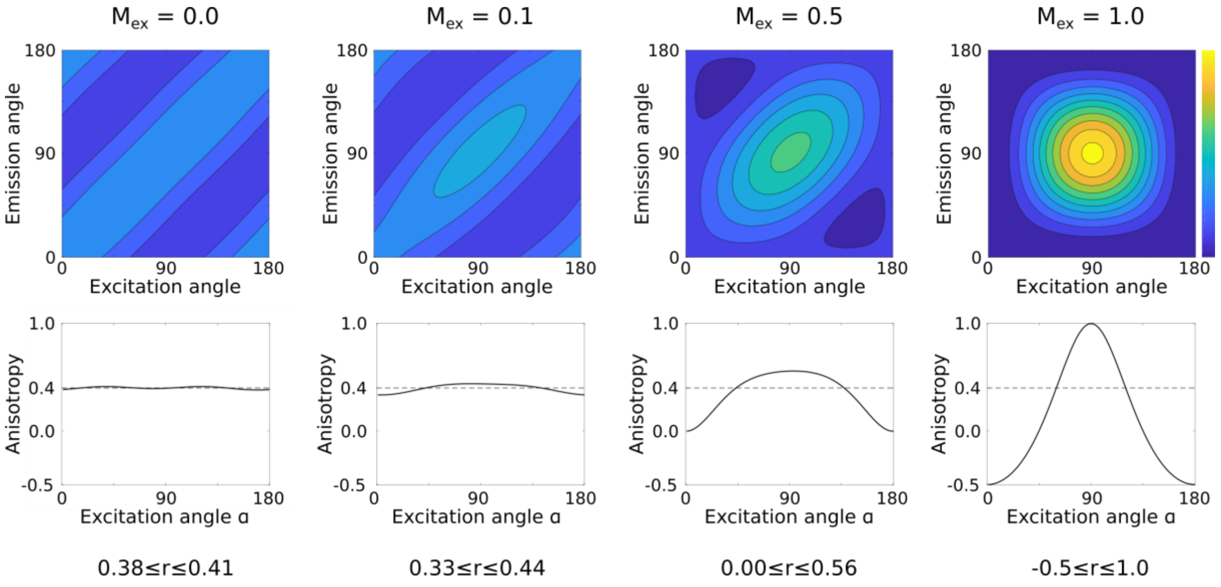

Supplementary Figure 2| Fluorescence anisotropy as a function of the chosen excitation polarization direction (α) – in silico. The ensemble of chromophores in the simulations is changed from anisotropic ($M_{ex} = 0$) to fully oriented ($M_{ex} = 1$), with its main orientation axis at 90 degrees. Top row presents the polarization portrait while the bottom row presents r(α). Note that generating an anisotropic sample ($M_{ex} = 0$) via our simulations is a difficult task. This is because we implement a numerical solution that can only yield truly isotropic samples for an infinite number of iterations. Therefore, the small variation of the anisotropy value with α observed for $M_{ex} = 0$ ($0.38 \leq r \leq 0.41$) is a numerical artifact – sampling error. Such artifacts become less important as the degree of alignment increases. The limiting case of a completely aligned sample, $M_{ex} = 1$, is relatively easy to simulate as all dipoles in the system have the same orientation and it is the result of sampling a delta function. Details on the simulations can be found in the Methods section of the main manuscript.

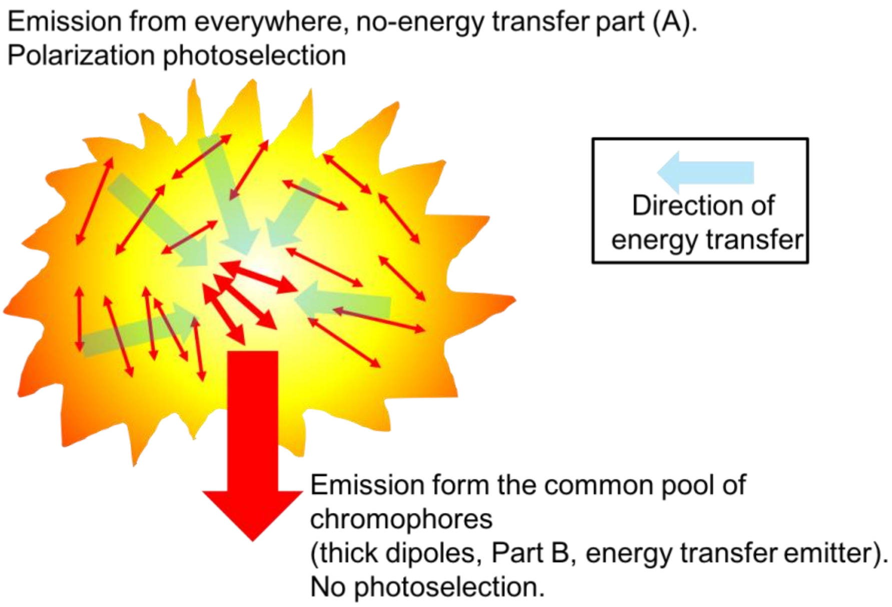

Supplementary Figure 3| Single funnel approximation. The common pool of chromophores (energy transfer emitter, thick dipoles) takes ε fraction of the total energy (part B). The remaining part (1- ε) is emitted from the initially excited chromophores (part A).

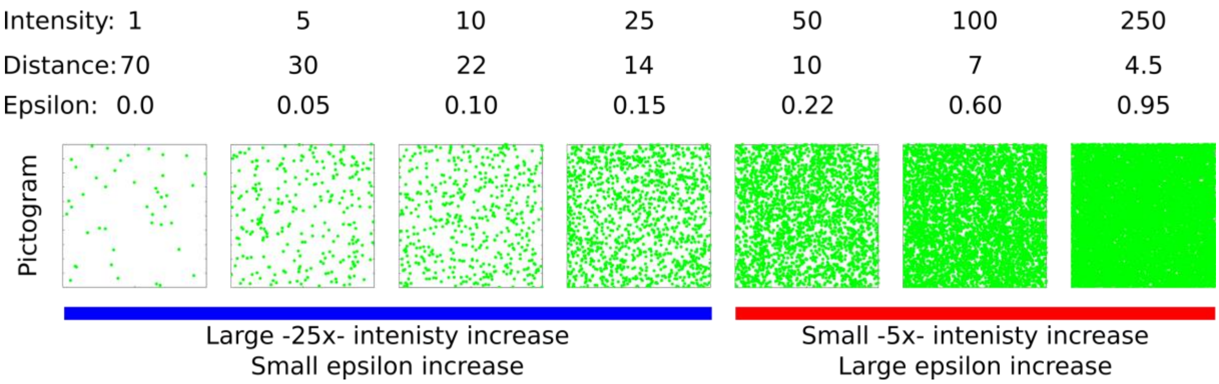

Supplementary Figure 4| Simple pictogram to illustrate the correlation between intensity and the energy funneling efficiency. The pictograms assume a random distribution of the labels in a 2D plane. Intensity values are given in arbitrary units, and distance in nanometers. Epsilon values are only approximate based on the results from our simulations as displayed on Figure 1 of the main manuscript. The size of each square pictogram is 1 micrometer. Since the energy funneling efficiency ε is sensitive to FRET, it is dependent on the distance between the fluorescent labels (FRET coupling). Fluorescence intensity of each square, on the other hand, is determined by the number of labels in the square. This means that while the density is low enough that the distance between labels is larger than 10 nm, then large intensity increments would induce only very small (or none) epsilon increments. Later, as the distance between labels approaches 10 nm, there will be a strong correlation between intensity and epsilon if we assume random distribution of the labels.

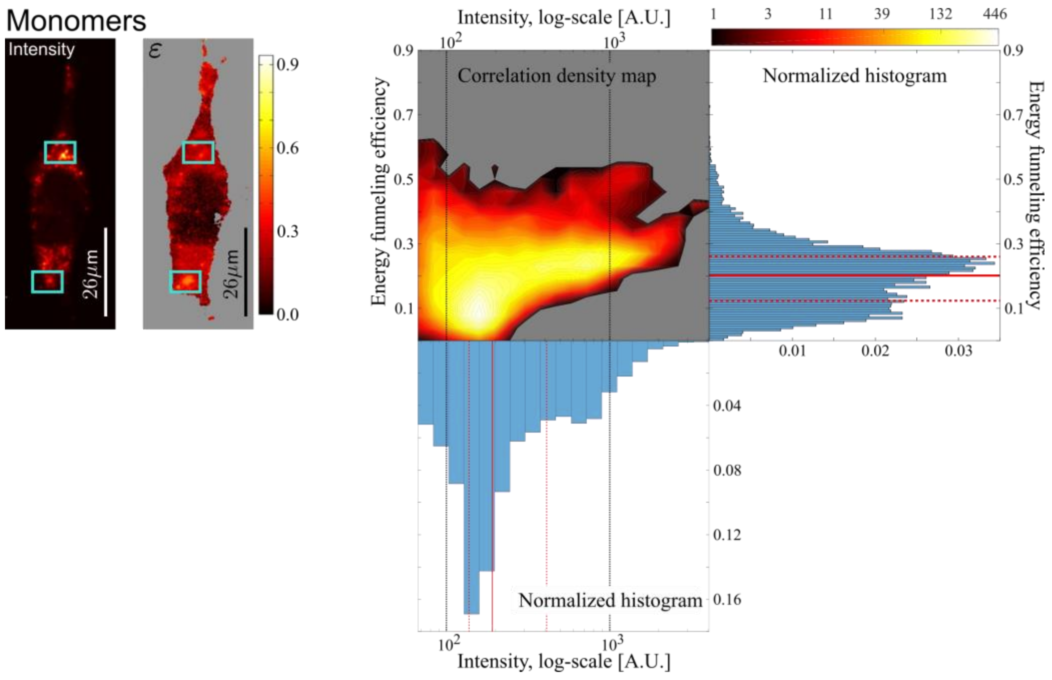

Supplementary Figure 5| Distributions and correlation between intensity and energy funneling efficiency for Figure 2-a of the main manuscript. Left: intensity and ε for monomers of α-syn labeled with ATTO-550 and incubated in MN9D cells. Right: Correlation density map and normalized histograms. Color bar of the correlation plot, and intensity axis follow a logarithmic scale, while the ε axis follows a linear scale.

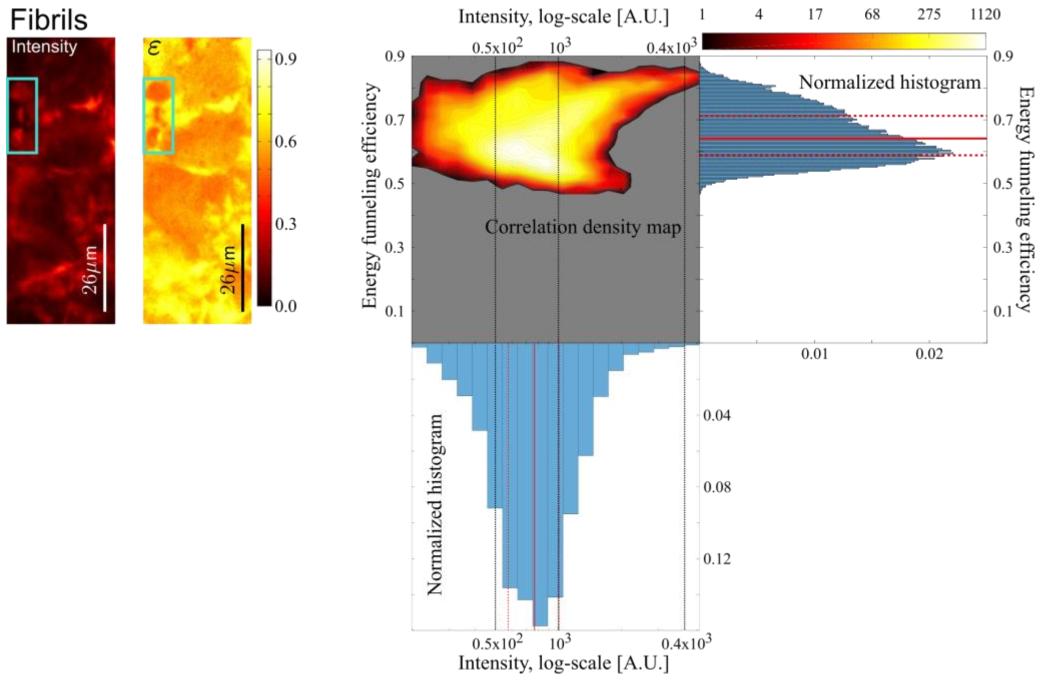

Supplementary Figure 6| Distributions and correlation between intensity and energy funneling efficiency for Figure 2-b of the main manuscript. Left: intensity and ε for fibrils of α-syn labeled with ATTO-550 and incubated in MN9D cells. Right: Correlation density map and normalized histograms. Color bar of the correlation plot, and intensity axis follow a logarithmic scale, while the ε axis follows a linear scale.

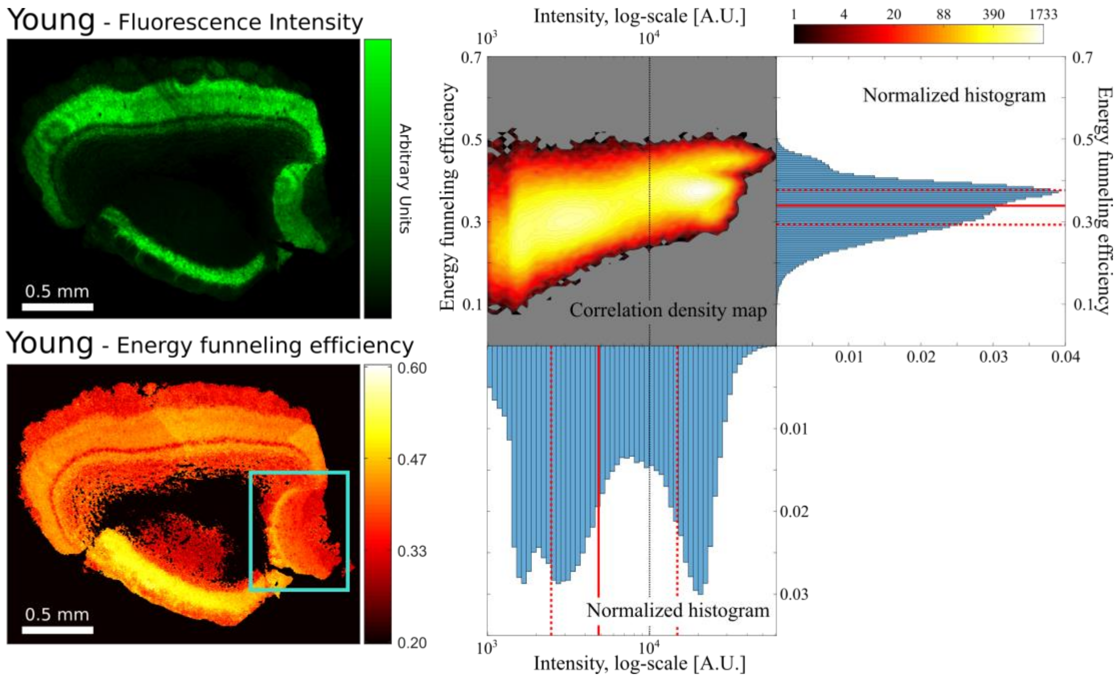

Supplementary Figure 7| Distributions and correlation between intensity and energy funneling efficiency for Figure 3-a, b of the main manuscript. Left: intensity and ε for the overview images of the olfactory bulb brain sections from a young mouse. The signal is generated by fluorescence of α-syn-GFP. Right: Correlation density map and normalized histograms. Color bar of the correlation plot, and intensity axis follow a logarithmic scale, while the ε axis follows a linear scale.

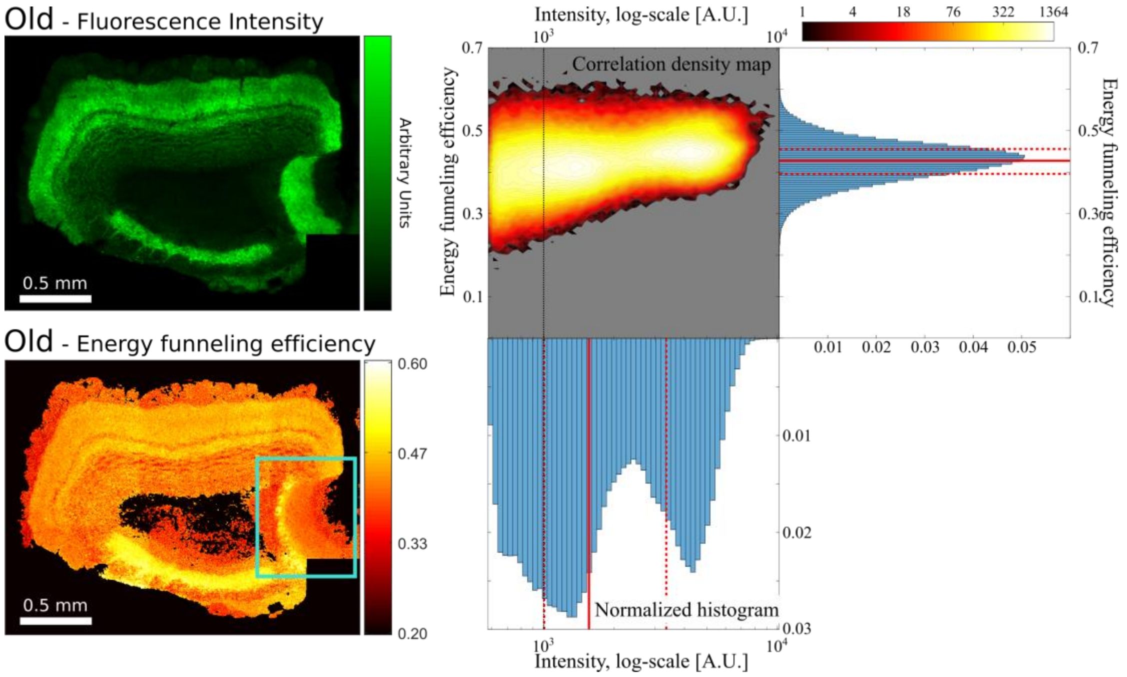

Supplementary Figure 8| Distributions and correlation between intensity and energy funneling efficiency for Figure 3-c, d of the main manuscript. Left: intensity and ε for the overview images of the olfactory bulb brain sections from an old mouse. The signal is generated by fluorescence of α-syn-GFP. Right: Correlation density map and normalized histograms. Color bar of the correlation plot, and intensity axis follow a logarithmic scale, while the ε axis follows a linear scale.

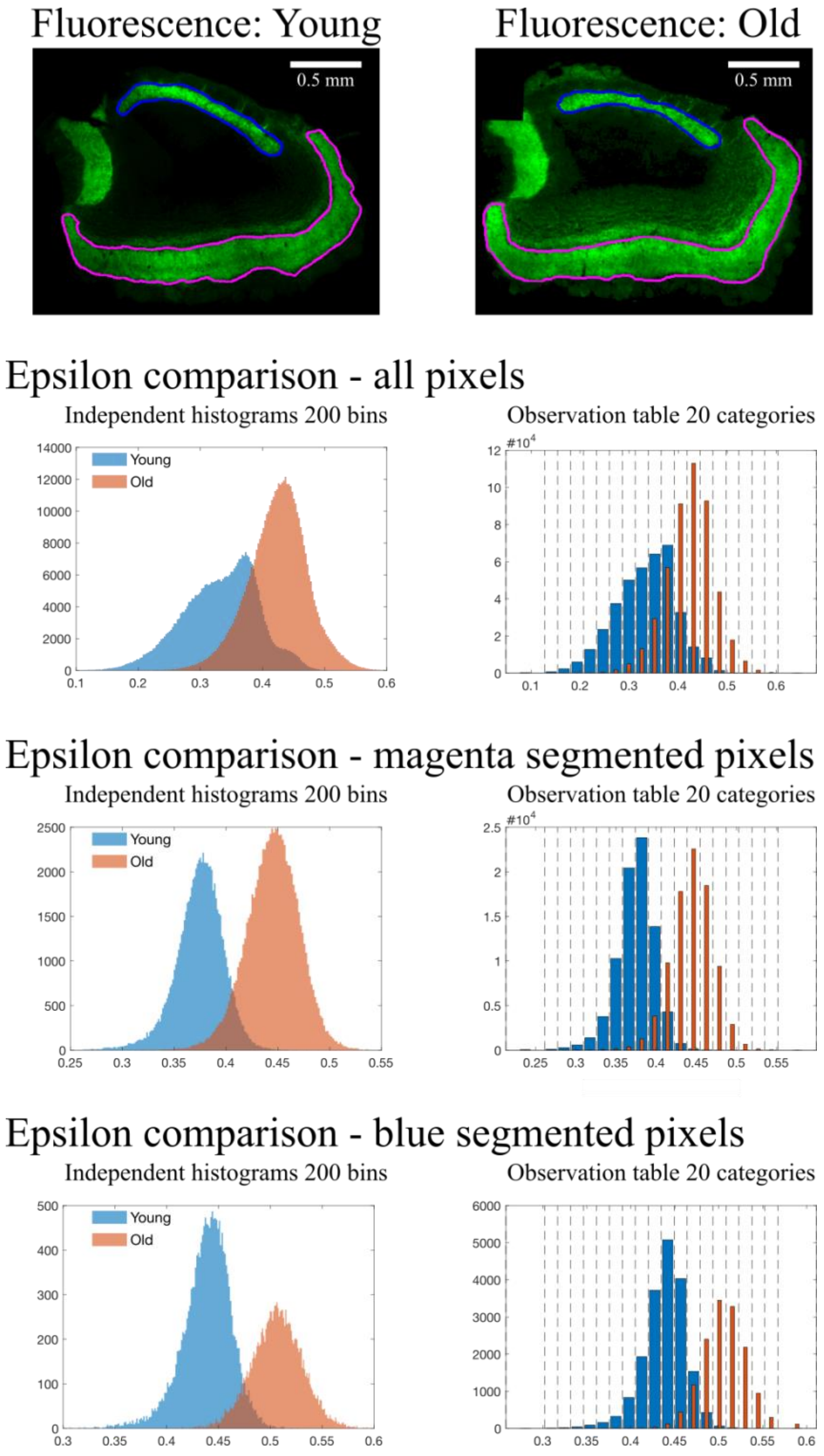

Supplementary Figure 9| Pearson's test of independence for the epsilon distributions for young and old mouse for Figure 3 of the main manuscript. Top: intensity images for the olfactory bulb brain sections from a young and old mouse. This is followed by the epsilon histograms for all pixels in the image, magenta segmented areas and blue segmented areas. As it can be seen both globally and locally, the epsilon values for the old mouse are significantly larger than those for the young mouse.

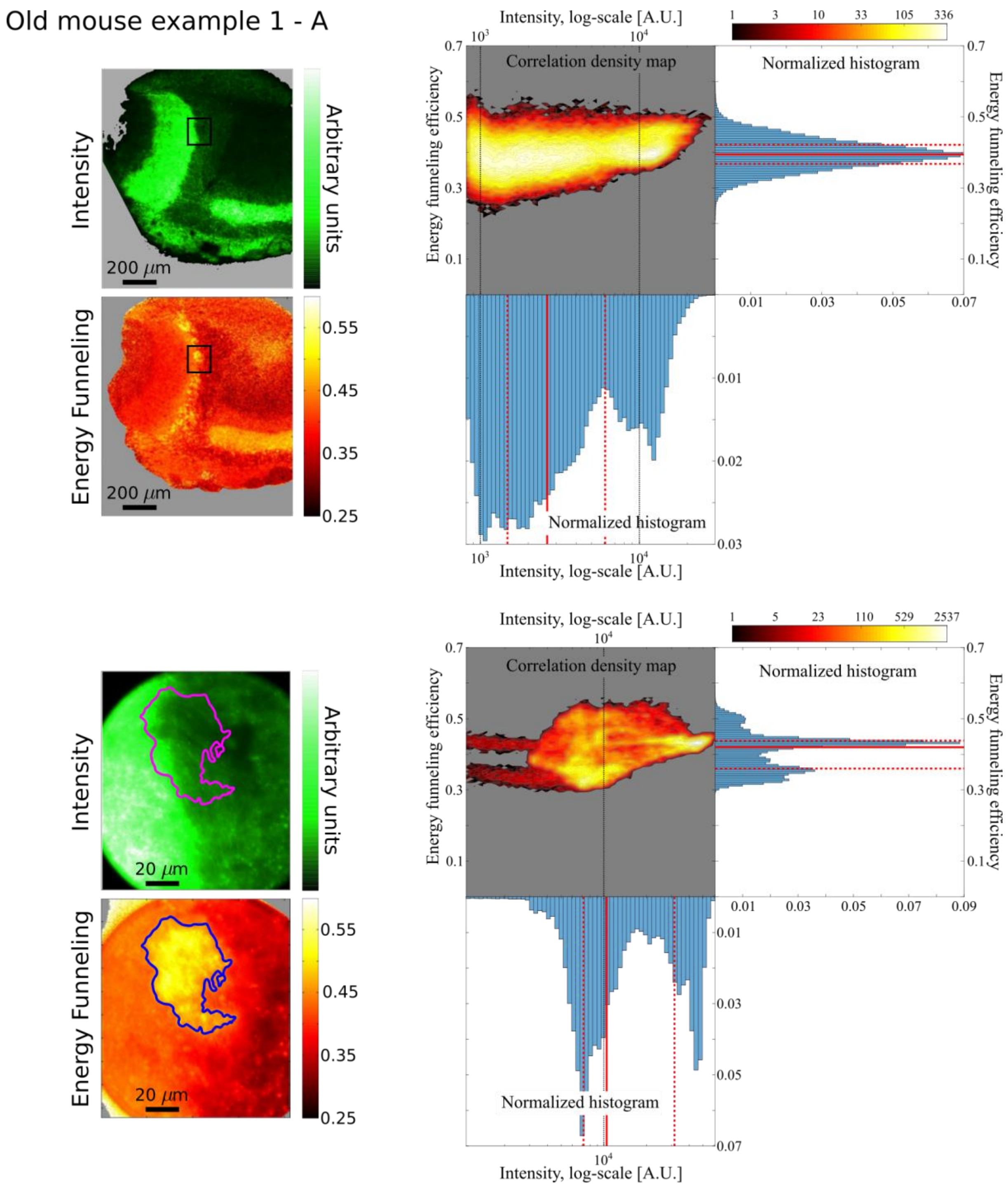

Supplementary Figure 10| Distributions and correlation between intensity and energy funneling efficiency for Figure 4-a of the main manuscript. Left: intensity and ε for overview images of the accessory olfactory bulbs (AOB) of an old mouse showing high expression of α-syn-GFP. Higher magnification images on the lower panel. The signal is generated by fluorescence of α-syn-GFP. Right: Correlation density map and normalized histograms. Color bar of the correlation plot, and intensity axis follow a logarithmic scale, while the ε axis follows a linear scale.

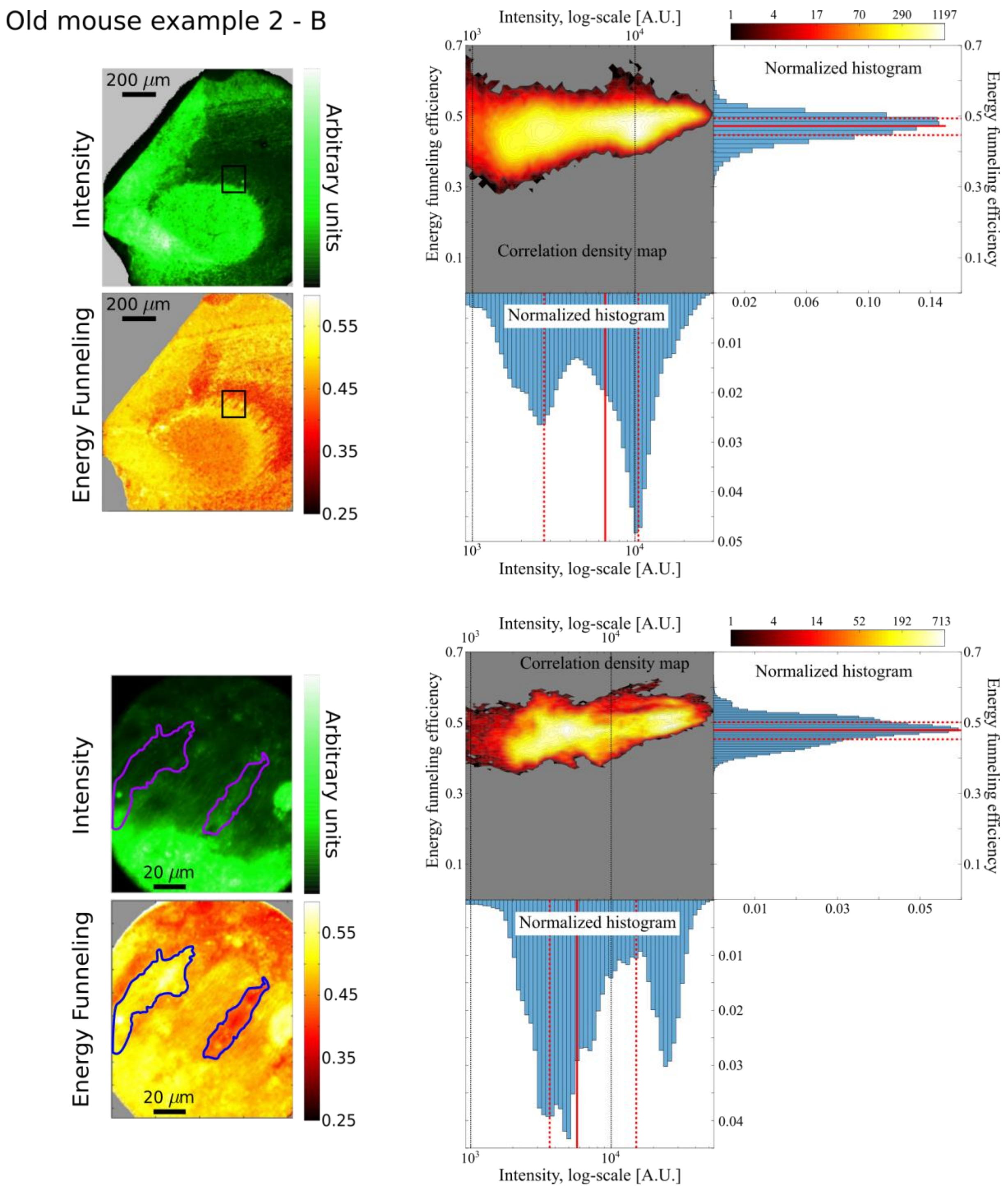

Supplementary Figure 11| Distributions and correlation between intensity and energy funneling efficiency for Figure 4-b of the main manuscript. Left: intensity and ε for overview images of the accessory olfactory bulbs (AOB) of an old mouse showing high expression of α-syn-GFP. Higher magnification images on the lower panel. The signal is generated by fluorescence of α-syn-GFP. Right: Correlation density map and normalized histograms. Color bar of the correlation plot, and intensity axis follow a logarithmic scale, while the ε axis follows a linear scale.

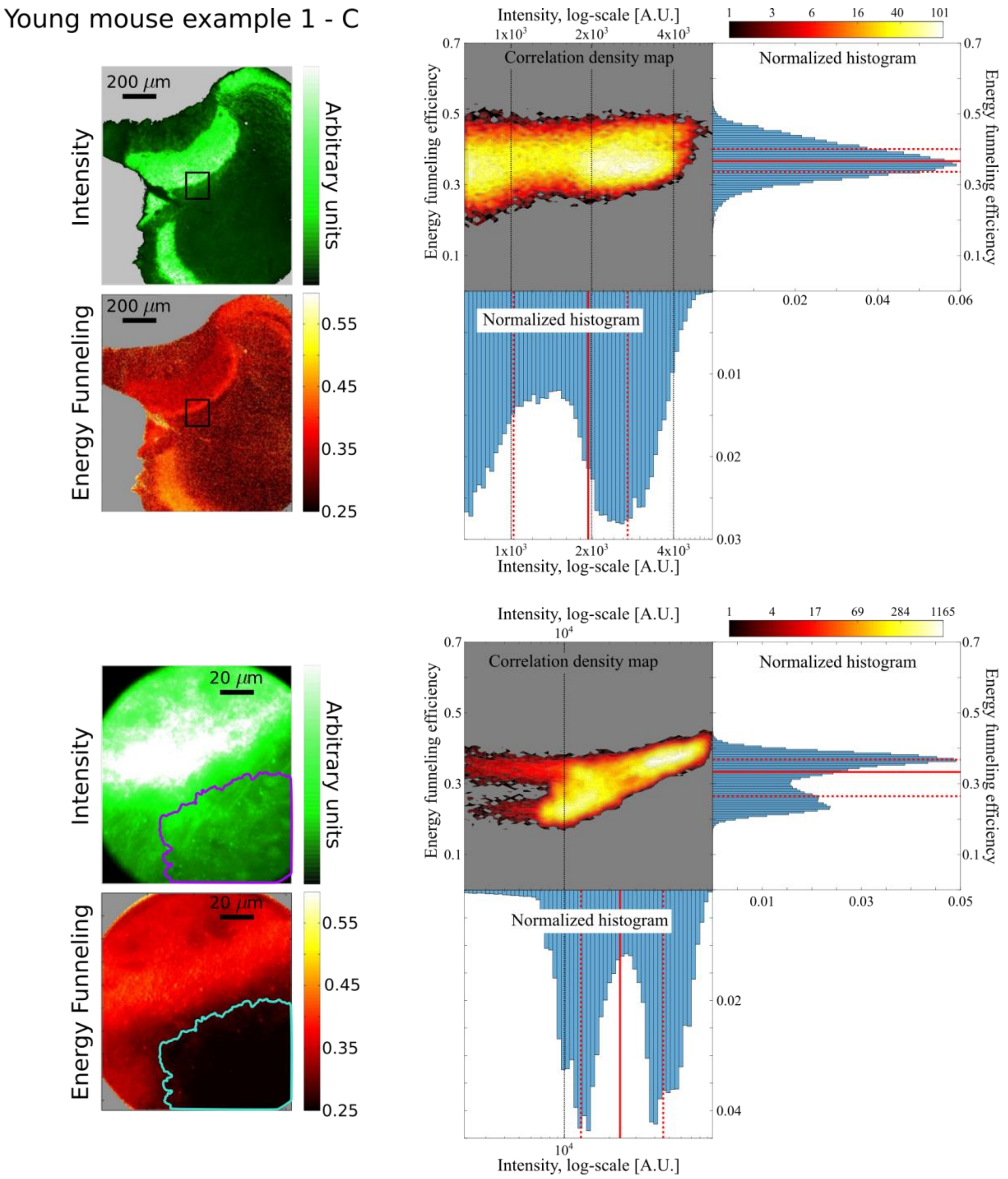

Supplementary Figure 12| Distributions and correlation between intensity and energy funneling efficiency for Figure 4-c of the main manuscript. Left: intensity and ε for overview images of the accessory olfactory bulbs (AOB) of a young mouse showing high expression of α-syn-GFP. Higher magnification images on the lower panel. The signal is generated by fluorescence of α-syn-GFP. Right: Correlation density map and normalized histograms. Color bar of the correlation plot, and intensity axis follow a logarithmic scale, while the ε axis follows a linear scale.

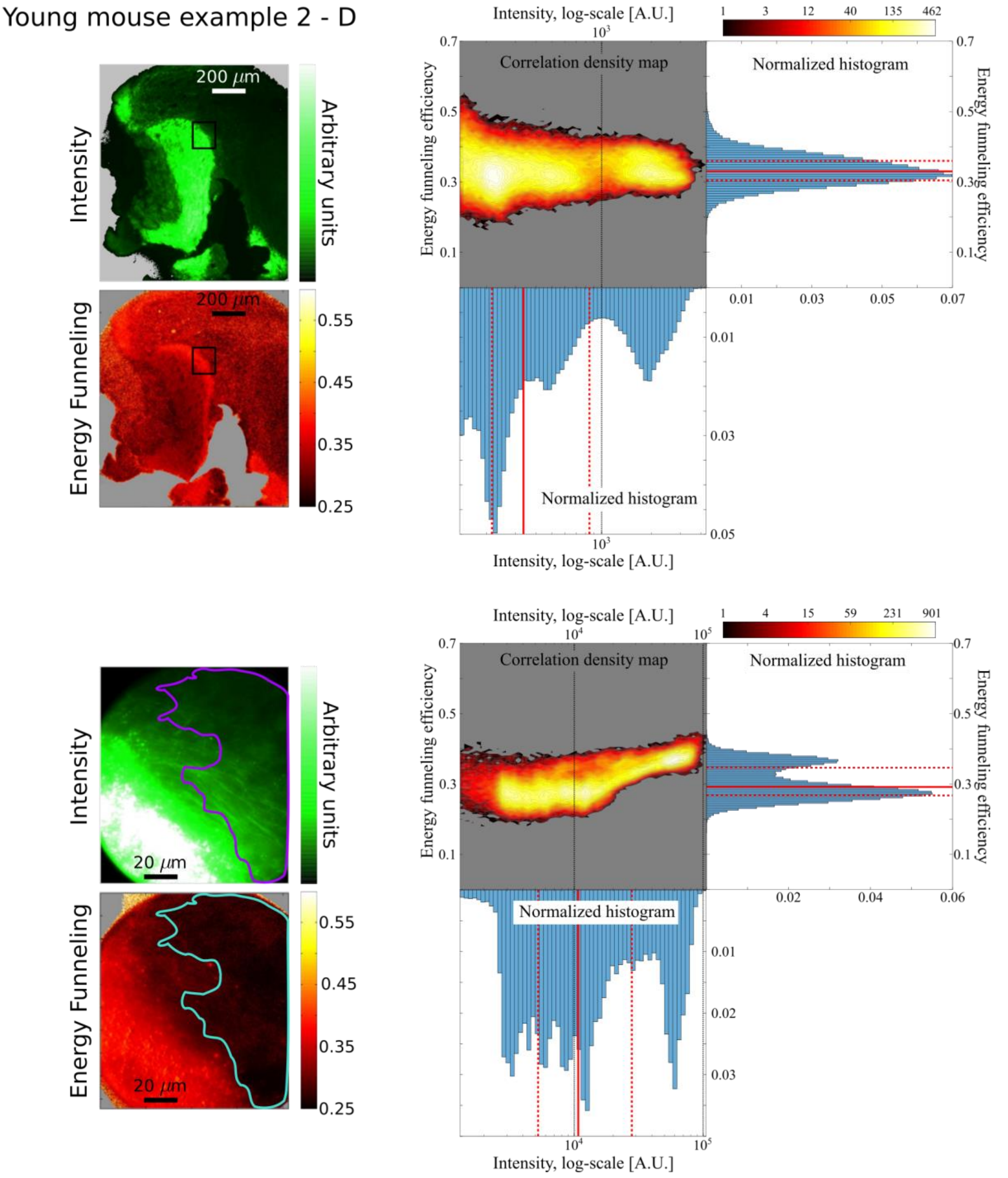

Supplementary Figure 13| Distributions and correlation between intensity and energy funneling efficiency for Figure 4-d of the main manuscript. Left: intensity and ε for overview images of the accessory olfactory bulbs (AOB) of a young mouse showing high expression of α-syn-GFP. Higher magnification images on the lower panel. The signal is generated by fluorescence of α-syn-GFP. Right: Correlation density map and normalized histograms. Color bar of the correlation plot, and intensity axis follow a logarithmic scale, while the ε axis follows a linear scale.

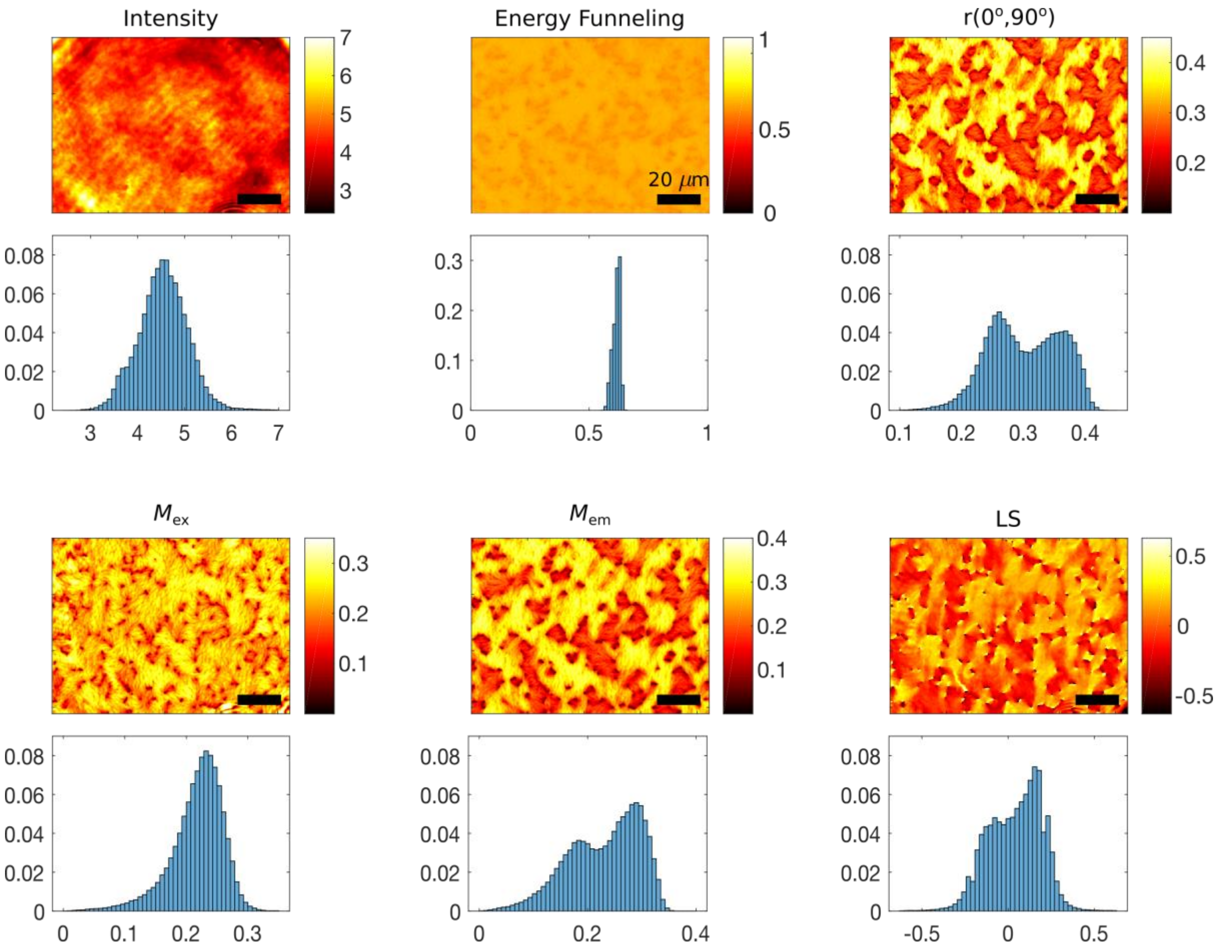

Supplementary Figure 14| 2D POLIM images and histograms for conjugated polymer pristine film. This film possesses an inhomogeneous structure in terms of the chain orientation at the scale of tens of micrometers, which can be seen as pronounced structures in the M_{ex} and M_{em} images. It is known from previous studies that the energy transfer efficiency within pristine conjugated polymer films is very high.[1] In accordance to this, the energy funneling parameter (epsilon) is large and shows only slight variation across the film (from 0.55 to 0.66, mean ε = 0.615, std = 0.016, coefficient of variation = 2.7%). On the other hand, due to preferential orientation of the polymer chains at the micro-scale, the variation in the fluorescence anisotropy (r) over the film is much more substantial (from 0.1 to 0.45, mean r = 0.302, std = 0.058, coefficient of variation = 20%). r exactly follows the structures observed in M_{ex} and M_{em} images.

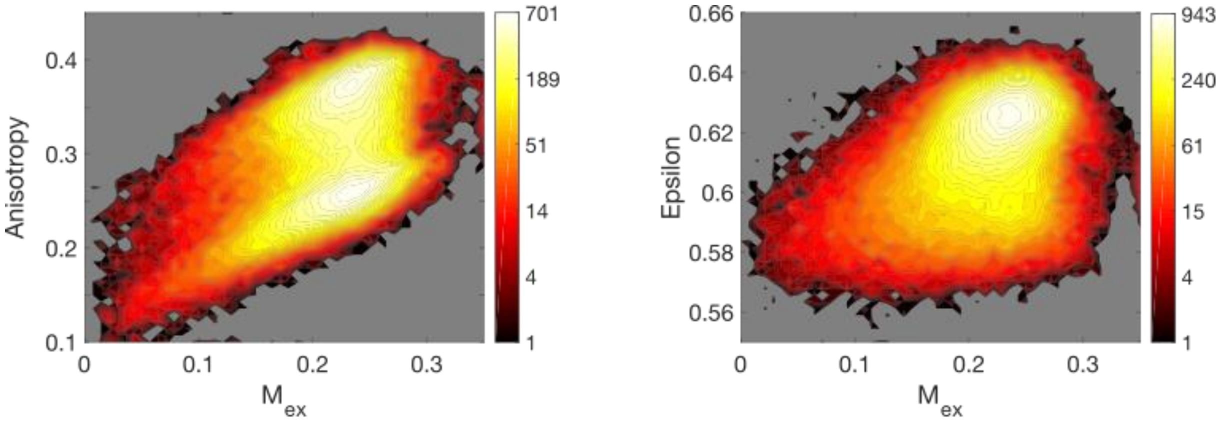

Supplementary Figure 15| Correlation between the sample's degree of orientation (M_{ex}) and the different FRET metrics (r and ε) in the conjugated polymer pristine film. Left: Correlation between M_{ex} and r. Right: Correlation between M_{ex} and energy funneling efficiency ε. As it can be seen the variation of ε with the sample's M_{ex} is significantly smaller. This is because, in this case, the fluorescence anisotropy does not work as a pure energy transfer contrast. Fluorescence anisotropy variations (contrast) are mainly due to chain orientation variations (M_{ex}), and not from energy transfer efficiency changes, as we know from photo-physics of conjugated polymers and see from the ε image.

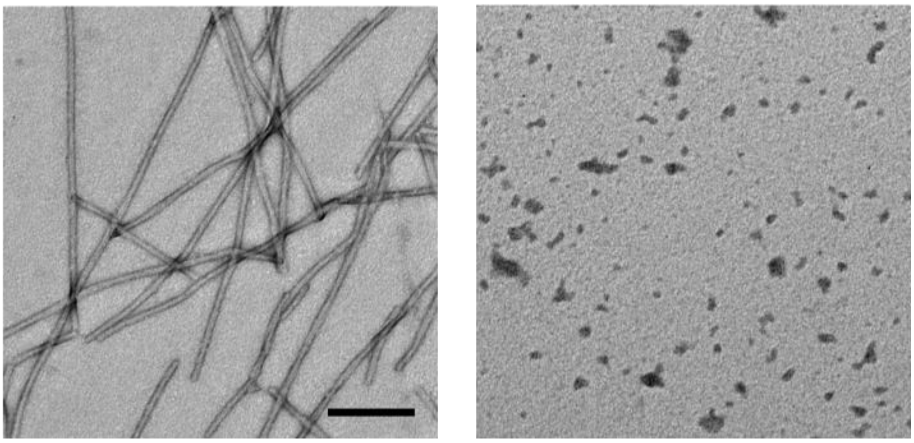

Supplementary Figure 16| Electron micrographs of α-syn fibrils and oligomers. Transmission electron images of negatively stained fibrillary (left panel) and oligomeric (right panel) α-synuclein used throughout this work. α-synuclein assemblies in PBS were adsorbed on carbon-coated 200 mesh grids and negatively stained with 1% uranyl acetate. The assemblies were imaged in a Jeol 1400 transmission electron microscope. The images were recorded with a Gatan Orius CCD camera (Gatan). Scale bar is 200 nanometers in length.

Supplementary Tables

Supplementary Table 1: Statistical descriptors of the energy funneling distributions for the data displayed on Figure 2 of the main manuscript.

Sample	Mean	Standard deviation	Median (Q50)	Minimum value	Quantile 25	Quantile 75	Maximum value
**Monomers**	0.199	0.097	0.202	$9x10^{-5}$	0.124	0.260	0.730
**Fibrils**	0.652	0.079	0.641	0.467	0.589	0.712	0.884

Supplementary Table 2: Statistical descriptors of the energy funneling distributions for the data displayed on Figure 3 of the main manuscript.

Sample	Mean	Standard deviation	Median (Q50)	Minimum value	Quantile 25	Quantile 75	Maximum value
**Young**	0.333	0.060	0.339	0.049	0.293	0.376	0.570
**Old**	0.425	0.047	0.427	0.140	0.396	0.456	0.682

Supplementary Table 3: Pearson's test of independence for the epsilon distributions for young and old mouse as shown in Supplementary Figure 9.

Comparison	x-value	Degrees of freedom	p-value
**All pixels**	3.9×10^5	19	<0.001
**Red segmented**	1.3×10^5	19	<0.001
**Blue segmented**	2.6×10^4	19	<0.001

Supplementary Table 4: Statistical descriptors of the energy funneling distributions for the data displayed on Figure 4 of the main manuscript.

Sample	Mean	Standard deviation	Median (Q50)	Minimum value	Quantile 25	Quantile 75	Maximum value
**a Old**	0.395	0.042	0.394	0.209	0.368	0.421	0.597
**a Old Zoom**	0.407	0.052	0.420	0.289	0.360	0.439	0.596
**b Old**	0.468	0.037	0.472	0.245	0.446	0.493	0.989
**b Old Zoom**	0.476	0.036	0.479	0.350	0.453	0.501	0.635
**c Young**	0.369	0.048	0.366	0.165	0.337	0.401	0.561
**c Young Zoom**	0.318	0.059	0.333	0.162	0.265	0.368	0.457
**d Young**	0.333	0.042	0.330	0.150	0.304	0.360	0.555
**d Young Zoom**	0.304	0.046	0.292	0.160	0.268	0.346	0.455

Supplementary Notes

Supplementary Note 1: Further details regarding 2D Polarization Imaging

Two-dimensional **Pol**arization **Im**aging (2D POLIM) can be seen as a further development of the fluorescence anisotropy and linear dichroism experiments. Its purpose is to characterize the correlation between the direction of the linearly polarized excitation light (direction of the exciting electric field in the sample plane) and the polarization of the resulting emission from the sample. Such information can be used to obtain information about:

a) Relative orientation of the absorbing and emitting dipole transitions, which in many cases can then be related to the physical structure of the object under study.
b) Excitation energy transfer (EET) processes, in particular this is one of the few methods sensitive to EET between energetically/spectrally similar chromophores, so-called homo-FRET.

Polarization portrait

In essence, 2D POLIM measures, in a wide-field configuration, the fluorescence intensity of the sample, **I**, as a function of the excitation polarization angle φ_{ex} and the orientation of the polarization analyzer φ_{em}. Experimentally, the sample is excited by linearly polarized light having its electric field parallel to the imaging plane, and at orientation (angular position) φ_{ex}. The fluorescence intensity is measured through an analyzer with transmission axis at angular position φ_{em}.[*] Therefore, the fluorescence intensity measured for each pixel in the imaging plane is a two-dimensional function, $\mathbf{I}(\varphi_{\text{ex}},\varphi_{\text{em}})$, called polarization portrait.

Modulation depth, modulation phase and luminescence phase shift

Integration of the polarization portrait over the excitation, φ_{ex}, or emission, φ_{em}, angle yields a one-dimensional function containing the modulation depth and modulation phase for fluorescence emission, M_{em} and θ_{em}, or excitation, M_{ex} and θ_{ex}, respectively:

$$\mathbf{I}(\varphi_{\text{em}}) = \int_0^{\pi} \mathbf{I}(\varphi_{\text{ex}},\varphi_{\text{em}})\, d\varphi_{\text{ex}} = \bar{I}(1 + M_{\text{em}}\cos(2[\varphi_{\text{em}} - \theta_{\text{em}}])) \qquad \text{Eq. 1}$$

$$\mathbf{I}(\varphi_{\text{ex}}) = \int_0^{\pi} \mathbf{I}(\varphi_{\text{ex}},\varphi_{\text{em}})\, d\varphi_{\text{em}} = \bar{I}(1 + M_{\text{ex}}\cos(2[\varphi_{\text{ex}} - \theta_{\text{ex}}])) \qquad \text{Eq. 2}$$

where, $\bar{I}$ is the average intensity value. M_{ex} and M_{em} describe the degree of orientation of the transition dipole moments, having values between 0 and 1, where 0 corresponds to an isotropic absorber/emitter (e.g. free rotating fluorescent labels in solution), and 1 corresponds to a linearly polarized absorber/emitter (e.g. single fluorescent label with fixed orientation). The modulation phases θ_{ex} and θ_{em} determine the angle of the main orientation axes of fluorescence excitation and emission, respectively.

The difference between the fluorescence excitation and emission modulation phases receives the name of luminescence phase shift:

$$\Delta\theta = \theta_{\text{ex}} - \theta_{\text{em}} \qquad \text{Eq. 3}$$

and indicates the presence of EET when its value is different from 0.

[*] Note that both φ_{ex} and φ_{em} are measured relative to the same axis, so-called laboratory axis.

Fluorescence anisotropy

Within the framework of the polarization portrait, the fluorescence anisotropy, r:

$$r = \frac{I'_{\parallel} - I'_{\perp}}{I'_{\parallel} + 2I'_{\perp}} \qquad \text{Eq. 4}$$

is calculated from two points of the 2D surface, namely:

$$I'_{\parallel} = I(\alpha, \alpha) \qquad \text{Eq. 5}$$

$$I'_{\perp} = I\left(\alpha, \alpha + \frac{\pi}{2}\right) \qquad \text{Eq. 6}$$

where, α is an arbitrarily chosen excitation polarization angle, which is often vertical in the laboratory coordinate framework. This yields the following expression of r:

$$r = \frac{I(\alpha, \alpha) - I\left(\alpha, \alpha + \frac{\pi}{2}\right)}{I(\alpha, \alpha) + 2I\left(\alpha, \alpha + \frac{\pi}{2}\right)} \qquad \text{Eq. 7}$$

Note that because the angle α is arbitrarily chosen the fluorescence anisotropy is uniquely defined only for isotropically absorbing samples ($M_{\text{ex}} = 0$). If this condition is not met, then the value of r will depend on α – the chosen excitation polarization direction. See Supplementary Figure 1 for a pictogram of the concepts discussed above.

Fluorescence anisotropy as a function of the excitation polarization for oriented samples

Supplementary Figure 2 illustrates how the selection of the reference excitation polarization angle α affects the fluorescence anisotropy calculated for systems of varying degree of alignment along 90° direction. The dipoles in this example do not rotate. To facilitate the interpretation of the plots, the distance between dipoles in the simulation was such that no EET occurred among them. Therefore, the fluorescence anisotropy values obtained are equivalent to the so-called fundamental anisotropy for a large ensemble of fluorophores, where the absorbing and emitting transition dipoles within each single fluorophore are parallel. If there was no preferential orientation, then the anisotropy should be 0.4.

As discussed in the main manuscript, for an isotropic sample, $M_{\text{ex}} = 0$, the fluorescence anisotropy is independent of the reference excitation angle, α. With increasing degree of alignment there is an increasing dependence of the anisotropy on α. Note that even for a very small degree of alignment, $M_{\text{ex}} = 0.1$, the anisotropy values vary significantly enough ($0.33 \leq r_{0.1} \leq 0.44$) to be detected experimentally and, thus, lead to imaging artifacts. This is because, for such oriented samples, the anisotropy value becomes a function of both local sample orientation and local EET efficiency. In such situations 2D POLIM will be preferable as the alignment/orientation and EET effects are effectively separated and displayed in different imaging contrasts. Local EET variations will appear in the energy funneling image, while local alignment/orientation variations will appear in the modulation depth and modulation phase images. For an experimental example see Supplementary Discussion - Fluorescence anisotropy vs energy funneling, experimental comparison.

Energy funneling efficiency - ε

Now the question is how we can extract an EET metric from the polarization portrait. Here we provide a short explanation while the full description can be found in the literature.[2–5]

The single funnel approximation (SFA) is a methodology used to characterize the so-called light harvesting (or energy funneling) properties of materials. The principle behind the SFA is to express

the polarization portrait as a linear combination of two components having a clear physical meaning: A-no energy transfer, B-energy transfer:

$$\mathbf{I}(\varphi_{\mathrm{ex}}, \varphi_{\mathrm{em}}) \approx (1-\varepsilon)\mathbf{A} + \varepsilon\mathbf{B} \qquad \text{Eq. 8}$$

The no energy transfer part of the luminescence signal **-A-** possesses the properties of an ensemble of non-rotating chromophores without energy transfer between them. Namely 100% photoselection, meaning that the polarization properties of the emission from **A** are dependent on the excitation polarization direction. For example, if the ensemble of chromophores giving rise to signal **A** absorbed light isotropically, then the fluorescence anisotropy of its luminescence would be 0.4.

The energy transfer component **-B-**, to the contrary, does not possess any photoselection. The polarization properties of the light coming from **B** are independent from the excitation polarization.

Before we go more to the mathematical description of the parameter epsilon, let us discuss the analogy of the SFA to the situation of hetero-FRET when the emission from the donors and acceptors can be measured separately due to the spectral shift between their emission. In this case the full luminescence intensity is a sum of the emission from the donors and acceptors:

$$\mathbf{I} = (1-\delta)\mathbf{I}_{\mathrm{Donor}} + \delta\ \mathbf{I}_{\mathrm{Acceptor}} \qquad \text{Eq. 9}$$

where, δ is the energy transfer efficiency from donors to acceptors.

As one can see, the two equations above (8 and 9) are exactly the same. The only difference is that in the case of homo-FRET we are not able to separate donors and acceptors by their spectra, and we need to do it by their polarization properties using photoselection. Therefore, if we would measure a sample with hetero-FRET (only hereto-FRET; no energy transfer between donors is allowed), the epsilon would be equal to the contribution of the acceptor emission to the total fluorescence.

In layman's terms, the SFA assumes that each measured chromophore transfers ε fraction of their total excitations to an EET-emitter, while $(1-\varepsilon)$ remains at the initially excited chromophores (Supplementary Figure 3). The EET-emitter is the energy acceptor of the system, which sometimes is referred to as the tip of the *energy transfer funnel*. This EET-emitter consists of a common pool of emissive states and is coupled to all absorbing states, directly or indirectly♠, via EET processes. Therefore, ε is called the *energy funneling efficiency*. It can be shown that such assumptions lead to the following master equations:[3,5,6]

$$\mathbf{A} = \widehat{N} \sum_{i=1}^{\widehat{N}} \cos^2(\varphi_{\mathrm{ex}} - \varrho_i)\cos^2(\varphi_{\mathrm{em}} - \varrho_i) \qquad \text{Eq. 10}$$

$$\mathbf{B} = \frac{\widehat{N}}{2}[1 + M_{\mathrm{f}}\cos(2[\varphi_{\mathrm{em}} - \theta_{\mathrm{f}}])]\widehat{N} \sum_{i=1}^{\widehat{N}} \cos^2(\varphi_{\mathrm{ex}} - \varrho_i) \qquad \text{Eq. 11}$$

where, the fluorescence emission of the EET-emitter has modulation depth M_{f} and orientation θ_{f}, and an unknown and complex N-dipole system (e.g. many fluorescent labels) is represented by a new group of $\widehat{N}$ standard dipoles with equal fluorescence excitation cross section♦. For a pictogram describing the single funnel approximation see Supplementary Figure 3.

It is very important to note that the EET-emitter can consist of more than one dipole transition. To better illustrate this point, consider the following two examples:

♠ by indirectly we meant that a transition dipole can be coupled to the EET-emitter via complex multi-step energy transfer processes.

♦ fluorescence excitation cross section: absorption cross section times the fluorescence quantum yield

1) Imagine the presence of a FRET antenna where a single red-absorbing label is used to attract the energy absorbed by a group of green-absorbing (red-fluorescent) probes. In an ideal case, the red-absorbing label will work as a common emitting site regardless of the initial location of the excited state in the probe system. Such EET-emitter would then be linearly polarized ($M_\mathrm{f} = 1$) and having an orientation θ_f equal to the orientation of the red-absorbing dipole.
2) Imagine the presence of a group of strongly coupled dipoles, such as in the case of closely packed fluorescent labels. For simplicity, assume that the coupling is such that once energy is absorbed by a member of the group this energy is freely transferred to any member of the group with large efficiency and equal probability. The result of such EET is that the whole group works as a common pool of emissive states, which will be responsible for the fluorescence of the group even in the extreme case when we selectively excite a single member of the group. Therefore, M_f and θ_f solely depend on the relative orientation of the dipoles within the group.

While the equations above are very general, they are still not defined enough to enable the computation of ε via the fitting of an experimental polarization portrait. To do this we must specify the number of standard dipoles assumed ($\widehat{N}$) and introduce some geometrical constrains to such system.[3,5] Once this is done, and using a so called 3-dipole-model we obtain:

$$\mathbf{I}(\varphi_\mathrm{ex}, \varphi_\mathrm{em}) \approx (1-\varepsilon)\mathbf{A} + \varepsilon\mathbf{B} \qquad \text{Eq. 8}$$

$$\mathbf{A} = \frac{1}{2+\Gamma}\{\cos^2(\varphi_\mathrm{ex} - [\theta_\mathrm{ex} - \varrho])\cos^2(\varphi_\mathrm{em} - [\theta_\mathrm{ex} - \varrho]) + \Gamma\cos^2(\varphi_\mathrm{ex} - \theta_\mathrm{ex})\cos^2(\varphi_\mathrm{em} - \theta_\mathrm{ex}) + \cos^2(\varphi_\mathrm{ex} - [\theta_\mathrm{ex} + \varrho])\cos^2(\varphi_\mathrm{em} - [\theta_\mathrm{ex} + \varrho])\} \qquad \text{Eq. 12}$$

$$\mathbf{B} = \frac{1}{4}[1 + M_\mathrm{ex}\cos(2[\varphi_\mathrm{ex} - \theta_\mathrm{ex}])][1 + M_\mathrm{f}\cos(2[\varphi_\mathrm{em} - \theta_\mathrm{f}])] \qquad \text{Eq. 13}$$

where, M_ex and θ_ex are determined via integration of the polarization portrait as explained above. ϱ, Γ, M_f, θ_f and ε are the variables to be fitted via a minimization algorithm. ϱ and Γ represent geometrical constrains to the 3 standard dipoles used to simplify equations 10 and 11. They have the following properties:

$$\varrho(\Gamma, M_\mathrm{ex}) = \frac{1}{2}\arccos\left[\frac{M_\mathrm{ex}(\Gamma+2) - \Gamma}{2}\right] \qquad \text{Eq. 14}$$

$$0 \leq \Gamma \leq 2\frac{(1+M_\mathrm{ex})}{1-M_\mathrm{ex}} \qquad \text{Eq. 15}$$

Supplementary Discussion

Supplementary Discussion 1| Correlation between fluorescence intensity and energy funneling

As mentioned in the main manuscript, the standard approach to visualize protein aggregation is to selectively label the proteins of interest with a fluorescent marker, followed by fluorescence imaging of the regions of interest, and then considering bright areas in the images as indication of aggregation. While useful and conceptually correct, this criterion is ambiguous as it cannot distinguish between high protein expression and aggregates (densely packed proteins at scale <10 nm). This ambiguity can lead to the erroneous assignment of the aggregated/non-aggregated status. As an alternative to improve the analysis of fluorescent images we suggest the use of 2D POLIM, whose FRET metric - ε – is, strictly speaking, independent of the total fluorescence intensity. Note, however, that this does not mean that there should not be any correlation between ε and fluorescence intensity (i.e. correlation but no causation). This correlation, if exists, is generated by the relationship between inter label distance (density, Supplementary Figure 4) and the fluorescence intensity.

Simple theoretical model

Supplementary Figure 4 shows that in a simple model without concentration quenching, ε and intensity are correlated in the regime where energy transfer starts to take place. This means that an intensity-based criterion can indeed work when the aggregation is strong and concentration quenching is absent. However, it also shows that for relatively low label densities increasing of their concentration, although obviously leading to higher fluorescence intensity, does not lead to any substantial change of ε. So, for "low" density of labels (no aggregation) high brightness cannot be used as reliable criteria for real aggregation.

Experimental examples

Here, we discuss the correlation between fluorescence intensity and energy funneling for all the 2D POLIM images presented in the main manuscript. As can be seen in the Supplementary Figures 5-13, there is a strong correlation between the recorded fluorescence intensity and the obtained ε parameter. This correlation is very clear, for example, in Supplementary Figures 5-6, and the bottom correlation plot of Supplementary Figure 12-13. Therefore, as expected by the simple theoretical model discussed before, there is a larger probability of having large ε values if the fluorescence intensity is large. Note that the images showing clear correlation belong to the samples where the aggregation of the labeled protein is not very strong (i.e. monomeric α-syn in cells and α-syn in brain sections of young mice). What we observe here is that accumulation of the labels in local structures brings some of them at a distance comparable with the Förster radius leading to some extent of energy transfer. This is in line with the situation illustrated in Supplementary Figures 4 and Figure 1 of the main manuscript.

However, there are many instances where this simple correlation does not hold, even for the same micrometer scale area of the sample. For clear examples see Supplementary Figures 6, 10 and 11 where objects of small fluorescence intensity can have very large ε values. This illustrates how the 2D POLIM methodology allows improving the detection of aggregation beyond the traditional fluorescence intensity-based methodology.

One of the possible reasons for anticorrelation between intensity and FRET is concentration quenching, which makes a dense aggregate less bright than a low-density cluster of the same number of labels. Even without concentration quenching, a low-density object smaller than the microscope's resolution (500 nm) can be much brighter than, say, a small dense aggregate containing ten times less

labels. Both objects will appear as diffraction limited spots (one bight and one dim) and only the dim spot (dense) will have large energy funneling efficiency.

Supplementary Discussion 2| Comparison between preformed fibrils and monomers of α-syn

A quantitative comparison (Supplementary Table 1) between the energy funneling distributions of the monomer and fibril samples clearly shows that fibrils present significantly larger ε values (0.65±0.08) than the monomers (0.2±0.1). The correlation plots between fluorescence intensity and ε (Supplementary Figures 5-6) demonstrate that for larger intensity values there is a larger probability of having large ε values. Interestingly, for both fibrils and monomers the ε distributions are far from normally distributed, and there are clear cases of anti-correlation between fluorescence intensity and ε values for the fibrils. The later indicates that not all fibrils are packed with equal density and that larger intensity values do not directly indicate more densely packed fibrils but can also easily arise from locally larger concentration (accumulation) of the fibrils. To the contrary, low intensity regions showing high ε values probably arise from dense aggregates with low fluorescence yield due to concentration quenching. The epsilon distribution for the monomer reports on small changes on the distance between monomeric α-syn at scales < 10 nm. It is quite remarkable that while the fluorescence intensity values span over more than one decade, the ε distribution has a relatively narrow distribution.

Supplementary Discussion 3| Changes in epsilon (aggregation) with mouse age

A quantitative comparison (Supplementary Tables 2-3) between the energy funneling distributions of the young (Supplementary Figure 7) and old (Supplementary Figure 8) mouse shows that ε values are significantly larger in the old mouse (0.43±0.05) than in the young mouse sample (0.33±0.06).

To test the effect of age (independent variable) in the observed epsilon values we employ a Person's test of independence on the epsilon distributions for old and young mouse (Figure 3 of the main manuscript). The reason for choosing the Pearson's test is that we do not have to assume any knowledge about the underlaying distributions. As presented in Supplementary Figure 9 and Supplementary Table 3, the test clearly shows that the epsilon distributions for the young and old mice are different, being larger for the older mouse. For the 3 comparisons done, namely all pixels and 2 sub-regions, the p-value of the test was always smaller than 0.001.

Higher magnification images of the accessory olfactory bulb (AOB) were obtained to investigate the differences between the aggregation pattern of young and old mice. As can be seen from Supplementary Figures 10-13, not only the overall value of the ε distribution becomes larger for the older mice, but also there are strong deviations from the intensity-ε correlation for the older mice, which are not clearly visible in the younger mice.

Supplementary Discussion 4| Fluorescence anisotropy vs energy funneling, experimental comparison

Here we discuss how the fluorescence anisotropy (FA) reports not only about variations in FRET but also on changes in the orientation/organization of the dipoles in the sample, while the energy funneling efficiency parameter ε demonstrates a much better-defined energy transfer sensitivity. This statement is illustrated by the data presented in Supplementary Figures 14 and 15.

Pristine films of conjugated polymers prepared as explained in Supplementary Methods result in films of uniform thickness, where the closely packed polymer chains are oriented to some degree at the micro scale (see small $M_{\mathbf{ex}}$ domains in Supplementary Figure 14). Due to these changes in orientation/organization the FA becomes an unreliable FRET reporter, as it cannot disentangle the

contributions from orientation and energy-transfer to the final FA value (Supplementary Figure 15, note the very different scale of FA and ε variations). On the other hand, ε remains large and fairly uniform as a result of the large and uniform energy transfer efficiency presented in the films, which has been predicted theoretically and demonstrated via numerous methods.[1]

Supplementary Discussion 5| Extra comments about homo-FRET simulations

In the main manuscript we discuss the influence of dimers (two chromophores located within the Förster radius) on the observed energy funneling parameter from an ensemble of chromophores containing a certain fraction of dimers. We mention that if there is a preferential collinearity between the two chromophores comprising the dimers, then the energy funneling increase due to the presence of dimers would be smaller than that presented in Figure 1-b of the main manuscript where no collinearity is assumed. The reasons behind this effect, while not trivial, are presented only here to help the readability of the main manuscript.

Let us consider the case where dimers are very far away from each other (right hand side of Figure 1-b of the main manuscript). In the case of preferential collinearity, the ε increase (anisotropy drop) will be smaller than that presented in the main manuscript where there is no correlation between the orientation of the chromophores in the dimer. Due to the extremely efficient energy transfer that occurs within a dimer, no matter which of the two chromophores is excited the energy will always be emitted with equal probability from any of them. Moreover, because 2D POLIM is a polarization-based measurement, it is only sensitive to EET that occurs between differently oriented dipoles. Any transfer between collinear dipoles cannot be assessed and, thus, it would be interpreted as a no-EET signal. Therefore, the increased collinearity between the chromophores of the dimer increases the amount of no-EET like signatures in bulk measurements, which then decreases the overall ε value.

Supplementary Methods

Conjugated polymer sample preparation: The film of a 1:1 blend of π-conjugated polymer poly[4,8-bis(5-(2-ethylhexyl)thiophen-2-yl)benzo[1,2-b:4,5-b′] dithiophene-2,6-diyl-alt-(5-(2-ethylhexyl)-4H-thieno[3,4-c]pyr-role-4,6(5H)-dione)-1,3-diyl] (PBDT-TPD) and poly[[N,N′-bis(2-octyldodecyl)-napht-halene-1,4,5,8-bis(dicarboximide)-2,6-diyl]-alt-thiophene-2,5-diyl] (PNDI-T) was prepared by spin casting at 1000 rpm of 12.5mg/ml solution in chloroform on 0.17 mm thick microscope cover slip. This resulted in 150 nm film thickness.[7]

Supplementary References:

1. Scheblykin, I. G., Yartsev, A., Pullerits, T., Gulbinas, V. & Sundström, V. Excited state and charge photogeneration dynamics in conjugated polymers. *J. Phys. Chem. B* **111,** 6303–6321 (2007).
2. Camacho, R., Thomsson, D., Yadav, D. & Scheblykin, I. G. I. Quantitative characterization of light-harvesting efficiency in single molecules and nanoparticles by 2D polarization microscopy: Experimental and theoretical challenges. *Chem. Phys.* **406,** 30–40 (2012).
3. Camacho, R. *et al.* Fluorescence polarization measures energy funneling in single light-harvesting antennas—LH2 vs conjugated polymers. *Sci. Rep.* **5,** 15080 (2015).
4. Camacho, R. *et al.* Polarization imaging of emissive charge transfer states in polymer/fullerene blends. *Chem. Mater.* **26,** 6695–6704 (2014).
5. Camacho, R. Polarization portraits of light- harvesting antennas: from single molecule spectroscopy to imaging. (Lund University, 2014).
6. Camacho, R., Thomsson, D., Yadav, D. & Scheblykin, I. Quantitative characterization of light-harvesting efficiency in single molecules and nanoparticles by 2D polarization microscopy: Experimental and theoretical challenges. *Chem. Phys.* **406,** 30–40 (2012).
7. Xu, X. *et al.* 8.0% Efficient All-Polymer Solar Cells with High Photovoltage of 1.1 V and Internal Quantum Efficiency near Unity. *Adv. Energy Mater.* **8,** 1700908 (2018).
